# The “Parahippocampal Place Area” Responds Preferentially to High Spatial Frequencies in Humans and Monkeys

**DOI:** 10.1371/journal.pbio.1000608

**Published:** 2011-04-05

**Authors:** Reza Rajimehr, Kathryn J. Devaney, Natalia Y. Bilenko, Jeremy C. Young, Roger B. H. Tootell

**Affiliations:** 1 Athinoula A. Martinos Center for Biomedical Imaging, Massachusetts General Hospital, Harvard Medical School, Charlestown, Massachusetts, United States of America; 2 McGovern Institute for Brain Research, Massachusetts Institute of Technology, Cambridge, Massachusetts, United States of America; University of California Davis, United States of America

## Abstract

Defining the exact mechanisms by which the brain processes visual objects and scenes remains an unresolved challenge. Valuable clues to this process have emerged from the demonstration that clusters of neurons (“modules”) in inferior temporal cortex apparently respond selectively to specific categories of visual stimuli, such as places/scenes. However, the higher-order “category-selective” response could also reflect specific lower-level spatial factors. Here we tested this idea in multiple functional MRI experiments, in humans and macaque monkeys, by systematically manipulating the spatial content of geometrical shapes and natural images. These tests revealed that visual spatial discontinuities (as reflected by an increased response to high spatial frequencies) selectively activate a well-known place-selective region of visual cortex (the “parahippocampal place area”) in humans. In macaques, we demonstrate a homologous cortical area, and show that it also responds selectively to higher spatial frequencies. The parahippocampal place area may use such information for detecting object borders and scene details during spatial perception and navigation.

## Abstract 2

A visual brain area that is thought to encode higher-level "place" information responds instead to lower-level "edge" information. A corresponding brain area is demonstrated in non-human species.

## Author Summary

Many reports suggest that different categories of visual stimuli are processed in correspondingly specific “modules” in the visual cortex. For instance, images of faces are processed in one cortical module (the “fusiform face area”), while images of scenes are processed in an adjacent module (the “parahippocampal place area,” or PPA). How does the PPA encode for such high-level, complex visual scenes? In this study, we show that at least part of the PPA response is due to a lower-level variable, reflected as higher spatial frequencies. These are prominent in the edges and details of scenes, but less prominent in faces and other stimuli. When we altered standard images of faces and places so that they only contained low, medium, or high spatial frequencies, we found that the PPA responded strongly to images containing high spatial frequencies. Importantly, using the same stimuli as for the human studies, we also demonstrated a homolog of human PPA in macaque temporal cortex (“mPPA”). As in humans, mPPA responds selectively to higher spatial frequencies. This demonstration of PPA in macaques paves the way for carrying out further electrophysiological and anatomical studies that may help elucidate the neural mechanisms for place selectivity in the human visual cortex.

## Introduction

There is lively interest in defining “category-specific” areas in the object processing regions of inferior temporal (IT) cortex, in both humans and monkeys. In such higher-order areas of the ventral visual pathway, discrete clusters of neurons reportedly respond selectively to specific categories of complex images such as faces [1]–[3], places [4],[5], body parts [6],[7], tools [8], animals [9], word forms [10], and perhaps even chairs [11]. However, in the campaign to map the category-specific regions of visual cortex, it is sometimes overlooked that stimuli of a common category often also share lower-level visual cues. Correspondingly, many cells in IT cortex are selective for specific lower-level properties, including surface curvature [12],[13], Fourier descriptor shapes [14], simple geometry [15],[16], non-accidental features (geons) [17], diagnostic features [18], color [19], and/or retinotopic location [20]. Thus, a hypothetical group of IT cells that responds robustly to a specific lower-level visual feature may also be interpreted as having a category-selective response—to the extent that these lower-level features are common to images from that category. For instance, functional magnetic resonance imaging (fMRI) studies have shown large-scale shape [21],[22] and eccentricity [23],[24] maps in human and macaque temporal cortex, which coincide with category-selective areas (e.g., face-selective patches). In fact, a recent theory suggests that overlapping continuous maps of simple features give rise to discrete modules that are selective for complex stimuli [25].

Here we demonstrate such a coincidence between a lower-level feature-selective map and category selectivity, in a prominent region of IT cortex known as the parahippocampal place area (PPA). Previously it has been reported that PPA responds more to images of “places” (or “scenes”) than to images from certain other object categories [4],[5]. However, place images encompass a virtually infinite range of possible visual stimuli: how could such a wide range of stimuli be coded in visual cortex? Similarly, how can “placeness” be quantified, to enable experimental study? Here we found that a simpler, lower-level feature selectivity contributes strongly to the higher-level place-selective response in PPA. This feature selectivity enhances the visual borders and edges of surrounding landmarks, which is useful in brain processing of places, scenes, and higher-order processes such as navigation.

## Results

**Figure 1:**
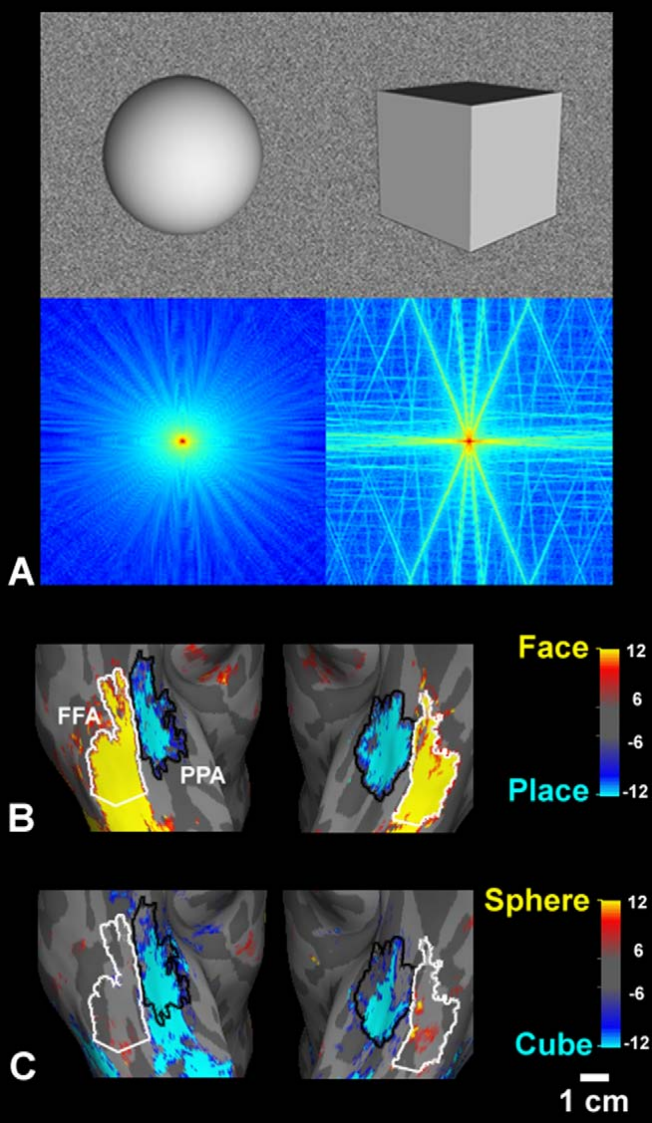
The higher-order cortical area PPA responds differentially to cubes versus spheres. (A) Sphere and cube stimuli and their FFTs. The top row shows sample images of the “smooth” sphere and the “smooth” cube. The bottom row shows the averaged 2-D FFT of the four sphere (left) and the four cube (right) images. The four images were distinguished only by changes in the location of the illuminant (see Materials and Methods). The red/blue color map represents the FFT magnitude (magnitude  =  red > yellow > cyan > blue) in Fourier space; the center of this space indicates the DC component of the FFT. The spectral distribution included a complex pattern of high SF components in the cube, but not in the sphere. Here and in the other FFT maps, the SF units are in cycles per pixel. (B) As a control, we produced an fMRI map based on a conventional, blocked-design comparison of naturalistic face versus place images, in five human subjects (using independent localizer scans). The group-averaged activity map is displayed on a ventral view of the averaged inflated cortical surface; the left hemisphere is shown on the right, with anterior towards the top. Faces and places produced relatively higher activity in FFA and PPA, respectively. (C) Relative activation to the cube versus the sphere is based on the averaged data, from the same five subjects shown in (B). The cube activated PPA robustly and selectively, with a topography similar to that produced by place-based activation. The color scale bars in the cortical maps here and in the other figures indicate the *p-*values, in a logarithmic format (i.e., −log_10_[*p*]).

The first sign of this lower-level selectivity arose serendipitously, when we were testing fMRI responses to very simple geometrical shapes including cubes and spheres. Figure 1 shows the cortical response to a cube relative to a sphere, when both stimuli were closely matched along lower-level dimensions other than shape. Surprisingly, we found that the cube activated PPA robustly and selectively—unlike the response in other visual areas (e.g., the adjacent, face-selective fusiform face area [FFA]).

This result has several implications. First, it revealed a vigorous and selective response to one computer-generated shape relative to another in PPA—though neither shape was a “place.” Second, this activity distinction occurred despite numerous similarities of the cube and the sphere: many other lower-level cues (e.g., surface reflectance, light source location, 3-D volume, and the visual field position of the shape center) were equated across the two stimuli.

What could explain this unexpected result? Among the remaining properties that distinguished these two shapes, the cube had distinct spatial discontinuities (edges and 3-D corners) in the foreground, whereas the sphere did not. These feature differences were reflected as a difference in fast Fourier transform (FFT), a quantitative global measurement of image properties: a complex pattern of high spatial frequency (SF) components was present in the cube, but not in the sphere (see Figure 1A). From this, it appears that PPA responses are modulated by spatial discontinuities in some form, perhaps reflected in the form of higher SFs.

**Figure 2:**
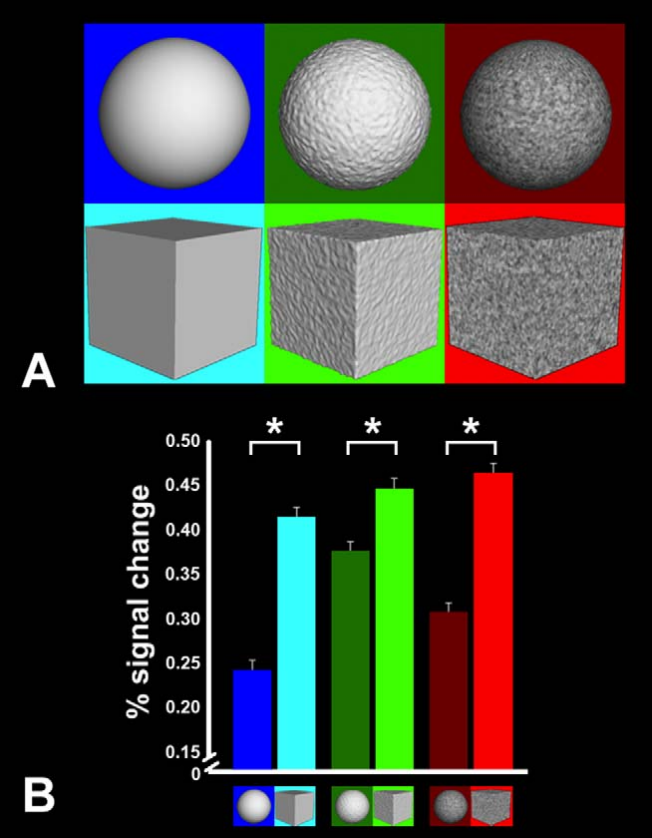
PPA responses to a range of computer-generated 3-D shapes. (A) In this experiment, the PPA responses were measured for “smooth,” “bumpy,” and “textured” versions of the sphere and cube (see icons). For each stimulus, the mean SF was estimated by the weighted averaging of SFs in the FFT power spectrum. This mean value was significantly greater in the three cubes, compared to the three spheres (*p*<0.005; paired *t*-test). (B) The fMRI responses to variations of the sphere and cube shapes in PPA. The PPA region of interest was defined functionally based on an independent place/face localizer (using naturalistic images; see Materials and Methods). An asterisk denotes a statistically significant difference (*F* = 14.45, *p*<0.05; ANOVA, Sidak post-hoc test). Error bars indicate one standard error of the mean, based on a within-subjects ANOVA design. PPA showed a consistently higher fMRI response to cubes (which had a higher mean SF) than to spheres, with a significant linear trend of increased activity (*p*<0.001) from the addition of bumpy and textured patterns to the stimuli. These results suggest a sensitivity to spatial discontinuities (e.g., higher SFs) in PPA, as confirmed more directly in subsequent experiments.

To further explore the effect of spatial discontinuities, we added either a white-noise texture (“textured”) or surface irregularities (“bumpy”) to the cube and sphere, and measured the fMRI responses independently to these stimuli, in addition to the original “smooth” sphere and cube, in common scan sessions. Again, we found that the responses in PPA were always higher for cubes than spheres, irrespective of the surface properties of these two shapes (Figure 2).

**Figure 3:**
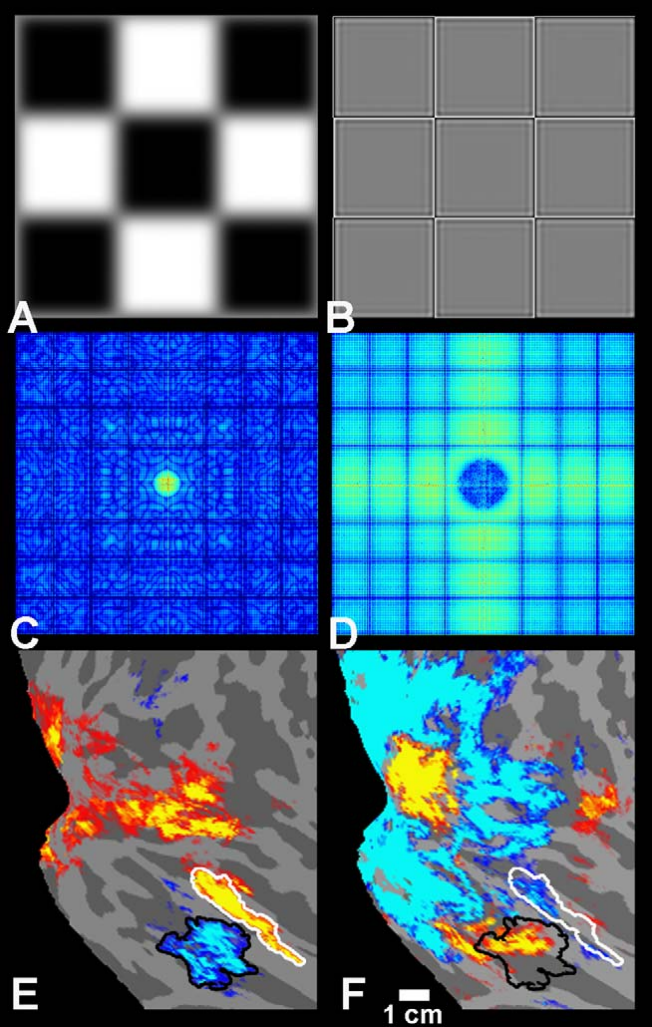
High-pass-filtered checkerboard images selectively activate PPA. (A and B) Examples of low SF (A) and high SF (B) checkerboards. In the actual experiment, an array of 6×6 checks was used, and the phase of the stimulus was systematically varied (see Materials and Methods for further details). The border around the stimuli is for illustration purposes only. (C and D) The FFTs of low SF (C) and high SF (D) checkerboards. The color map represents the FFT magnitude in Fourier space (see Figure 1A for more details about FFT maps). (E) The comparison between faces and places (an independent localizer scan) showed the classical areas FFA and PPA in the averaged map of four human subjects. The group-averaged activity map is displayed on a flattened view of the right occipito-temporal cortex. (F) The comparison of activity between high SF (yellow/red) and low SF (cyan/blue) checkerboards revealed a high SF bias within PPA. If anything, the opposite bias was found in parts of FFA. The maps are significant at the threshold of *p*<10^−2^.

Additional experiments revealed that even simpler, 2-D stimuli (classical flickering checkerboards) could selectively activate PPA, when those stimuli included high spatial frequencies. Cortical maps (Figures 3, S1, and S2) and region-of-interest analysis (Figure S3) showed a robust bias for high spatial frequencies in PPA, in response to checkerboard patterns—though such stimuli were neither places, nor objects, nor even 3-D shapes. The PPA response to high frequency checkerboards was even higher than that to normal, unfiltered checkerboards (see Figure S2)—suggesting that the presence of lower spatial frequencies in the normal image can effectively reduce the PPA response to high spatial frequencies, perhaps due to nonlinear inhibitory interactions (see Discussion).

**Figure 4:**
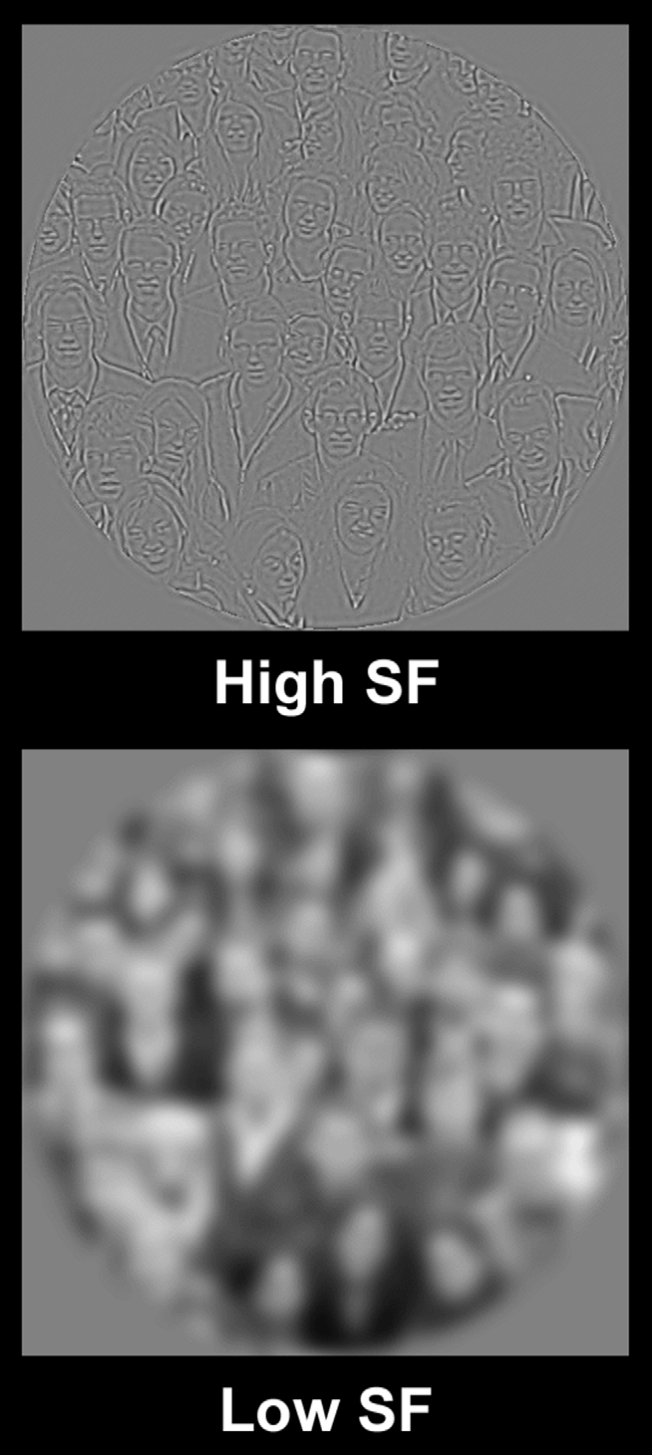
SF-filtered stimuli. The original stimuli were naturalistic face (and place) images (i.e., unfiltered). These stimuli were then spatially filtered using FFT to produce low SF (<1 c/deg), middle SF (1–5 c/deg), or high SF (>5 c/deg) images. For a complete set of stimuli, see http://nmr.mgh.harvard.edu/~reza/SFstimuli.pdf.

These results raise the question of whether at least part of the PPA response to “places” in previous reports may reflect the effect of uncontrolled spatial factors, including high spatial frequencies. To re-test this idea in the originally reported context, we first presented a range of naturalistic place and face images, because such place versus face comparisons have been used as a basic “localizer” for PPA (e.g., [26]). Intermixed with these normal stimuli, we presented the same set of images, after spatially filtering those images for low, middle, or high SFs. The initial SF-filtered images were generated based on arbitrarily chosen cut-off frequencies of 1 and 5 cycles/degree (c/deg) for low-pass and high-pass filtering, respectively (Figure 4). Since normal face and place images had different SF distributions (Figure S4A), the cut-off frequencies were shifted for place images, so that the resultant SF-filtered places had the same fraction of total power as the SF-filtered faces (see Figure S4 for further details). Outside the scanner, the effect of SF filtering on image perception was behaviorally assessed in each subject. All subjects could easily distinguish all SF-filtered images as either faces or places.

**Figure 5:**
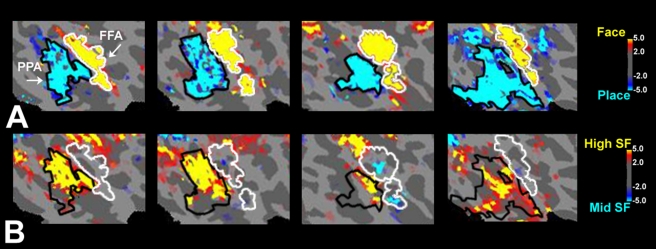
High-pass-filtered face images activate human PPA selectively. (A) The comparison of responses to normal faces versus normal places showed FFA and PPA in human ventral occipito-temporal cortex. Activation maps are displayed on magnified, flattened views of four hemispheres from four subjects. (B) The comparison of responses to high SF faces versus middle SF faces produced a selective activation to high SFs in PPA, even though the stimuli were faces instead of places. In some subjects, the reverse pattern of activation was found in the adjacent FFA.

**Figure 6:**
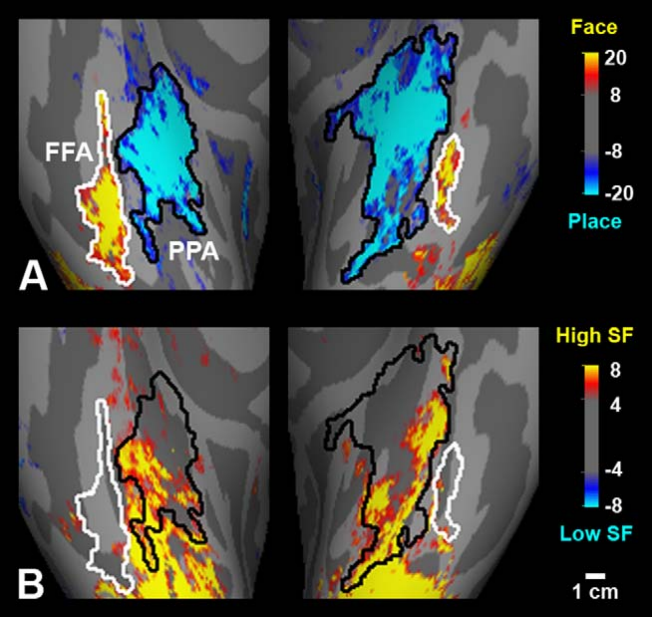
High-pass-filtered place images also activate human PPA selectively. (A) The comparison of responses to faces versus places showed FFA and PPA in the averaged map of four human subjects. The group-averaged activity map is displayed on a ventral view of an averaged inflated cortical surface; the left hemisphere is shown on the right. (B) The comparison of responses to high SF places versus low SF places revealed a high SF bias within PPA, in the averaged map of the same four subjects. The high SF patch was prominent in the lateral-posterior subdivision of PPA. As a control, FFA did not show any differential activity for SF variation.

Again, we found that PPA was especially responsive to “high SF” stimuli, compared to “middle SF” and “low SF” stimuli. This preferential response to high SFs was present for both face (Figures 5 and S5) and place (Figures 6 and S6) stimuli. In fact, the PPA response to *faces* (“nonoptimal” stimuli in PPA) almost doubled relative to that for normal images, when low and middle SF components were removed (see Figure S5). Maps of this high SF bias even reflected the same idiosyncratic topography as classically defined PPA (localized using normal, naturalistic images of places versus faces) in each subject (see Figure 5)—thus emphasizing the underlying biological link between PPA and its high SF bias. Interestingly, the high SF face images were as effective as the place images in activating PPA (Figure S7).

As an expected control result in the lower-tier (occipital) visual cortex (e.g., in areas V1, V2, and V3), the SF sensitivity co-varied systematically with the retinotopic representation of visual field eccentricity; a preference for higher SFs was clearly shown nearer the foveal cortex (Figure S8; see also [27],[28]). The foveal representation in area V8/VO [29],[30] may also have a preference for high SFs; however, our maps confirmed that the high SF activity in PPA was located anterior and ventral to V8/VO (Figure S9).

**Figure 7:**
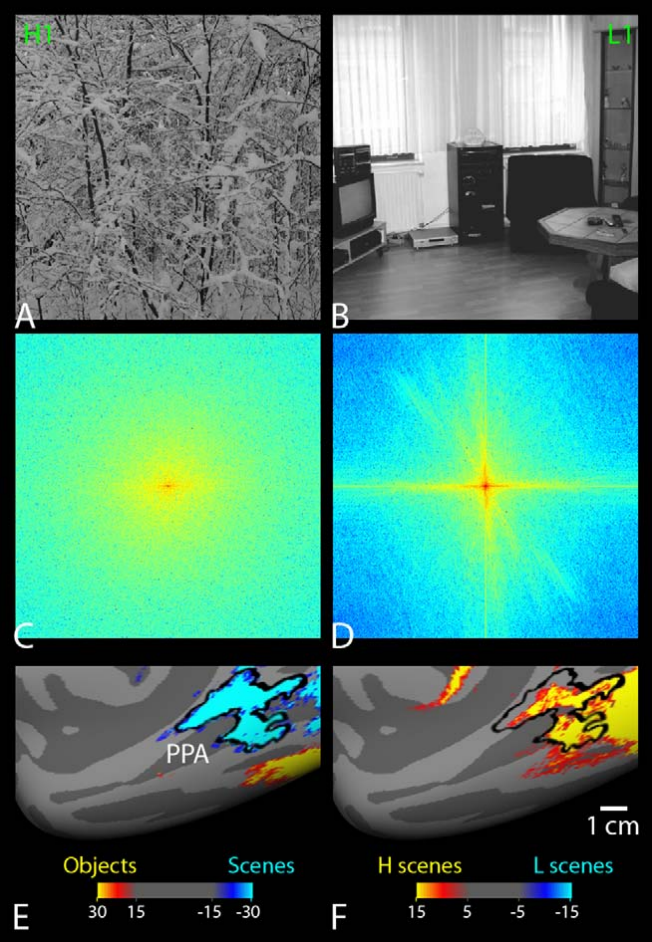
PPA activity reflects the spectral variation in natural scenes. (A and B) Examples of scenes with relatively higher (H scenes) (A) and lower (L scenes) (B) power at high SFs. These two images had the highest (H1) and the lowest (L1) normalized high SF power in our scene image database (see Figure S10 for details). (C and D) The FFT of the H1 (C) and L1 (D) images. The H1 scene had more high SF power (and less low SF power) than the L1 scene. The frequency bias was not confined to specific scene categories (e.g., outdoor versus indoor scenes). (E) PPA was localized based on a blocked-design comparison between scene stimuli and single-object stimuli, in five human subjects. The group-averaged activity map is displayed on a medial view of an averaged inflated cortical surface (right hemisphere). (F) The comparison between H scenes and L scenes revealed a strong fMRI activity for H scenes within PPA. This activity extended posteriorly, in occipito-temporal cortex.

In the experiments above, the SF content of the images was manipulated by frequency filtering. However, natural scenes can also vary dramatically in their SF distributions [31]. Does PPA activity reflect this spectral variation in normal (unfiltered) natural scenes? To answer this, we conducted an additional experiment comparing the fMRI response to normal scenes that were relatively dominated by either high or low SFs (Figures 7 and S10). Although both types of images were from the same semantic category (scenes), PPA (as defined by a scene/object localizer) responded significantly more to the scenes that had more power at high SFs (see Figure 7). This result indicates that intrinsic SF components in natural scenes can strongly modulate the PPA activity, even during natural scene viewing per se.

If this spectral sensitivity reflects a fundamental mechanism for neural scene coding in PPA, presumably it should also exist in a region of macaque visual cortex that corresponds to human PPA. To our knowledge, no such mechanism of high SF sensitivity has been reported previously in monkey. In fact, a monkey homolog of the human “place” area (PPA) has not yet been demonstrated. To test for a homolog of PPA in monkeys and for high SF sensitivity in this homolog area, we presented the same set of normal and SF-filtered stimuli to awake fixating monkeys, using fMRI procedures very similar to those used in the human scanning (see Materials and Methods).

**Figure 8:**
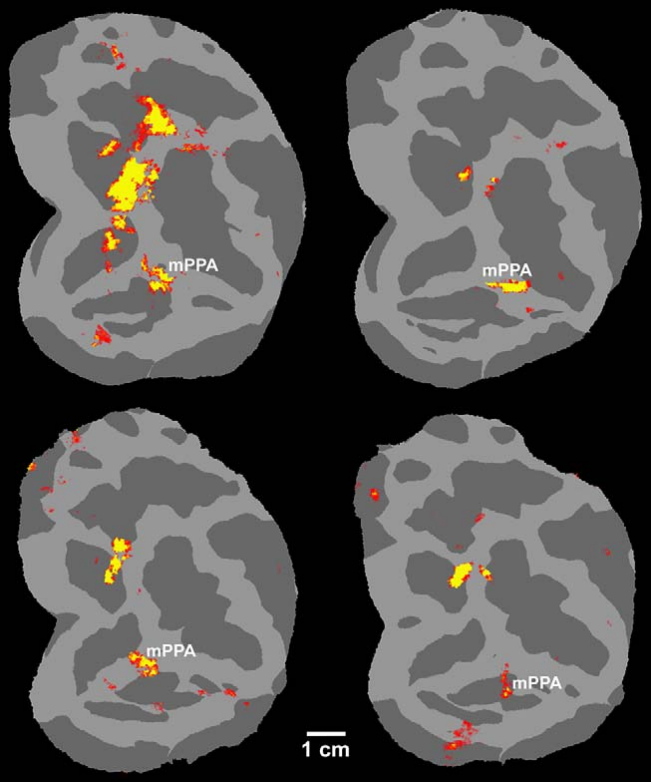
Evidence for a monkey homolog of PPA in individual maps. The maps show the activity produced by place images, in a comparison of places versus faces (map threshold: *p*<10^−4^), in awake monkeys. The activity map is displayed on a flattened view of macaque visual cortex in two monkeys (four hemispheres). The left panels show right hemispheres. The right panels show left hemispheres, mirror-reversed for ease of comparison. In all hemispheres, a place-selective patch (mPPA) was consistently localized in posterior IT cortex, adjacent/overlapping the posterior middle temporal sulcus, immediately ventral to the posterior temporal face patch (not shown). The topographic location of mPPA was slightly variable across individuals. A similar individual variability has been reported for the location of peak activity in human PPA ([49] and unpublished data). An additional place-selective patch was also localized in the anterior bank of the lunate sulcus, in occipito-parietal cortex. Presumably this dorsal patch is the monkey homolog of an additional human place-selective area located in the transverse occipital sulcus.

**Figure 9:**
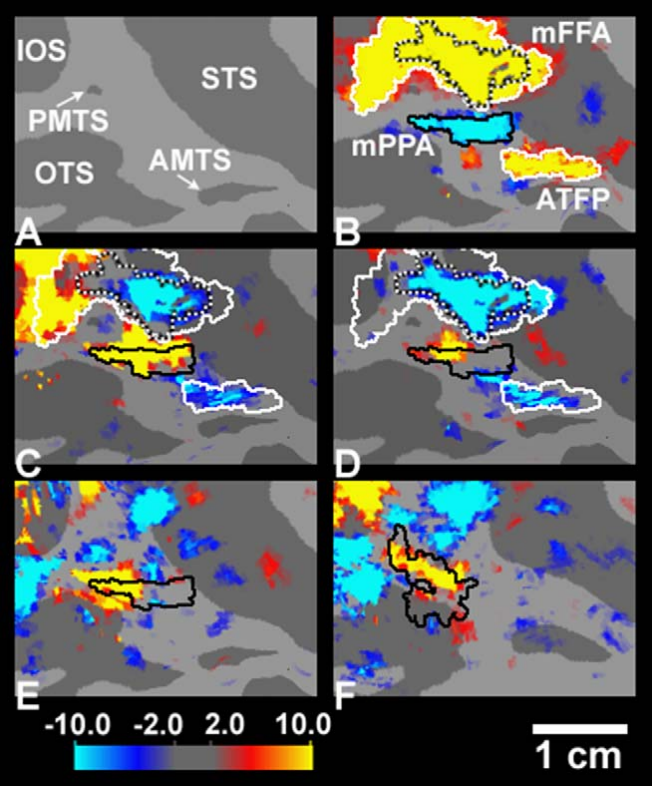
Topographic relationship between SF selectivity and place/face selectivity in macaque IT cortex. (A) A flattened view of macaque IT cortex (left hemisphere); posterior-to-anterior is left-to-right in this figure. Major sulci in this view are the following: AMTS, anterior middle temporal; IOS, inferior occipital; OTS, occipito-temporal; PMTS, posterior middle temporal; STS, superior temporal. (B) The activity map for normal faces (yellow) versus normal places (cyan) in the same hemisphere. As described earlier (e.g., [2],[33]), we consistently found a large face patch (here termed mFFA, based on its apparent homology with human FFA) in the fundus and the lower bank of superior temporal sulcus, and a smaller “anterior temporal face patch” (ATFP) in the upper bank of anterior middle temporal sulcus. Our maps also revealed a robust place-selective patch, mPPA (see also Figure 8, the upper-right panel), located adjacent and anterior-ventral to the mFFA. (C and D) The activity maps for high SF (yellow) versus low SF (cyan) places (C), and high SF (yellow) versus middle SF (cyan) faces (D) in the left hemisphere. The mPPA (especially its dorsal and posterior subdivisions) responded selectively to high SFs, whereas the face-selective mFFA (especially the central “hot spot” area of mFFA, which is most face-selective) responded selectively to lower SFs; a similar selectivity was found in the anterior face-selective area (anterior temporal face patch). That hot spot center of mFFA (delineated with a black/white dotted contour) was selected by increasing the statistical threshold to *p*<10^−16^ in the face/place map shown in (B). These SF-related activations were independent of the stimulus category (e.g., the “place” patch was preferentially activated even with high SF faces). (E and F) The activity maps for high SF (yellow) versus low SF (cyan) checkerboards in the left (E) and right (F) hemispheres. Again the mPPA in both hemispheres (marked with a black contour) showed a selective activation to high SFs.

First, using the original set of normal, naturalistic stimuli, we demonstrated a place-selective region in macaque IT cortex (Figure 8) that was located in a consistent location, immediately ventral to the largest IT face patch (Figures 9B and S11). Thus, this place-selective region in macaques is located exactly where it is predicted to lie, based on (1) the maps of face- and place-selective areas (FFA and PPA) in humans and (2) the previously reported location of the monkey homolog of FFA (mFFA) in macaques [2],[32],[33]. This monkey homolog of PPA (mPPA) was confirmed in an averaged map from three monkeys tested with additional control stimuli (Figure S12). All this evidence demonstrates that macaque monkeys do have a homolog of cortical area PPA, as defined originally in humans.

Importantly, subsequent tests showed that the mPPA was strongly selective for high SFs—again like PPA in humans (Figures 9C–9F and S13). In fact, the SF bias here was strong enough to reveal a striking double dissociation: relative fMRI activation in mPPA/mFFA could be effectively reversed by changing *either* object category (place versus face) *or* SF (high versus low) (see Figure 9). This finding also reemphasizes the lower-level nature of the trigger features in this “place processing” region of IT cortex: it is unlikely that monkeys have the same higher-order place associations as humans, in response to the same naturalistic images.

## Discussion

**Figure 10:**
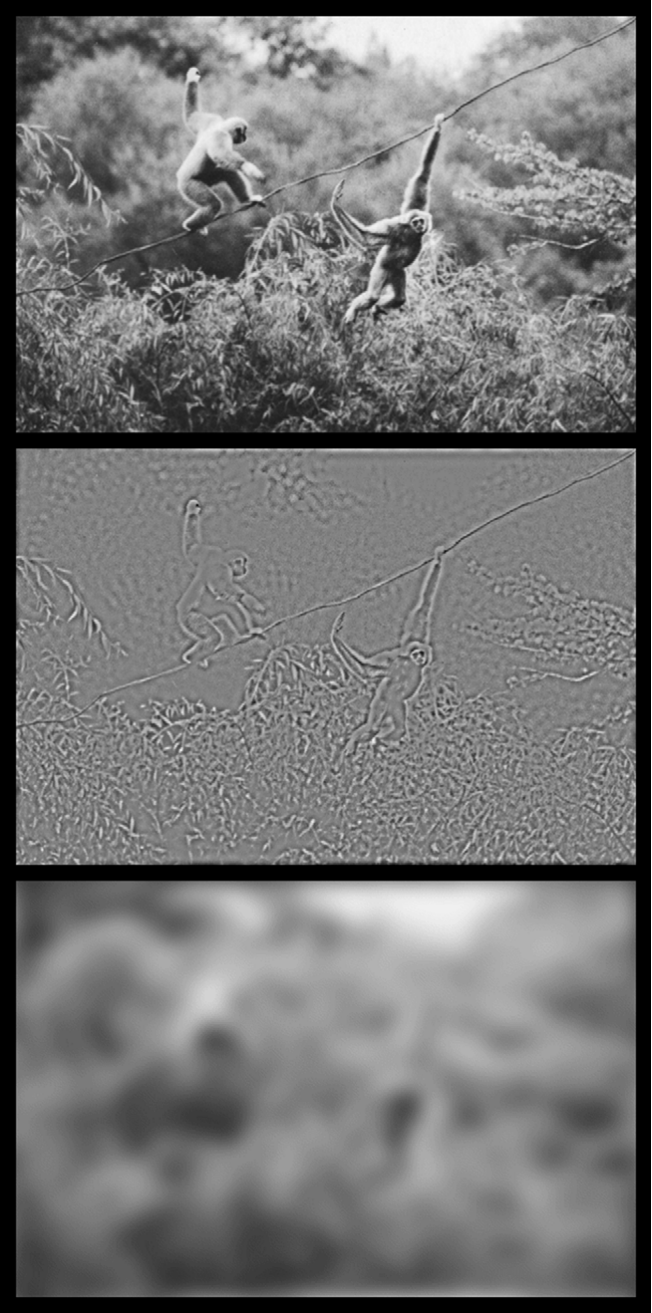
Detection of high SFs is crucial for primate place perception. The top panel shows an image of two apes (gibbons) navigating through the forest canopy. The middle and bottom panels show the same image, when filtered for high and low SFs, respectively. The high SFs highlight the borders of the branches and vines, but those borders are essentially invisible in the low SF image. In the normal image (top), the borders are arguably less identifiable than in the high SF image, due to competing visual clutter. The ability to exactly localize the borders of these environmental features has obvious survival value. The original image was taken from [50] with permission.

Why would this high SF bias exist in PPA? From a “top-down” perspective (e.g., a category-based model), this kind of information might be especially useful in defining “places” because high SFs, which are emphasized in edges, borders, and small spatial details, are crucial for survival in primate arboreal navigation (in reaching and grasping during brachiation [Figure 10], avoiding obstacles, etc.) or when detecting predators in visually complex surroundings. By this idea, a sensitivity to spatial discontinuities (as reflected in high SFs) may have been enhanced in PPA during evolution, in the service of spatial perception and navigation. This view integrates the data here with the “place-selective” model of PPA function.

However, our data also raise an alternative, “bottom-up” possibility (e.g., a lower-order interpretation). If a sensitivity to spatial discontinuities (e.g., a high SF bias) explains *some* of the PPA response, can an improved understanding of this lower-level spatial selectivity eventually explain *all* of that response? Our evidence clearly indicates a spatial selectivity in PPA, but this initial spatial characterization based on linear SF analysis is likely imperfect. Even in primary visual cortex, many cells show nonlinear inhibitory interactions in response to two SFs [34],[35]. Most commonly, such single-unit interactions consisted of lower SFs inhibiting higher frequencies. Our fMRI data showed analogous interactions in PPA (e.g., Figure S2). Thus, a more refined spatial characterization (beyond simple SF filtering) may ultimately explain even more of the variance in the PPA response. For instance, measurements of SF per se did not capture all of the variation in the PPA response in the cubes-and-spheres experiment. Ultimately, it may turn out that PPA responds selectively to a specific type of spatial discontinuity (e.g., corners, or Y-junctions), but not to others (e.g., linear edges)—though all these features add power at higher SFs in the FFTs from the entire image.

How can the evidence for a high SF bias be reconciled with the previously reported response to conventional place/scene images in PPA? In fact, there may be little or no discrepancy. As one might suspect, an FFT analysis (Figure S14) confirms that conventional (unfiltered) place images (e.g., images of buildings) have substantially more energy at high SFs than images of faces. Thus, both “high SF” and “place” hypotheses predict a robust activation in PPA in response to conventional place images.

According to previous reports (e.g., [36]), “intact” (normal) place images evoke a larger response in PPA than “scrambled” object images. In fact, this comparison is sometimes used as a conventional localizer for PPA. Intuitively, one might expect that the scrambled images would have more high SF power, because additional edges are added to the image during scrambling. Does this finding violate our conclusion that PPA responds best to higher SFs? To resolve this, we measured the normalized high SF power (the high SF power divided by the total power) for place and scrambled images used recently by others [36]. Counterintuitively, we found that the place images had more high SF power than the scrambled images (*p*<0.00001)—presumably reflecting the lower spatial density in the latter.

Previous human fMRI studies by Malach and colleagues [23],[24] showed that PPA responds better to peripheral stimuli than to foveal stimuli, relative to the neighboring FFA. It is also known that psychophysical sensitivity to higher SFs decreases at increasing eccentricities [37],[38]. Thus, our finding of a high SF bias in PPA appears to conflict with the peripheral bias in PPA. However, this apparent contradiction does not challenge our present results when all details are considered. In the experiments here, the full range of SFs was readily visible, at all eccentricities stimulated. In the fovea, human observers can resolve SFs up to ∼50 c/deg [38]; this is a log unit higher than the maximum cut-off frequency used in our experiments (i.e., >5 c/deg). Even at the peripheral edge of our stimuli (10° eccentricity), human observers can resolve SFs more than twice as high as the 5-c/deg cut-off frequency [38]. Thus, even the “high” SFs presented here were all “low” enough to be clearly visible (and thus accessible to PPA), even in the periphery of our stimuli. Nonetheless, given the previous reports of retinotopic biases in PPA [23],[39], it is conceivable that the strength of high SF bias in PPA varies with corresponding variations in retinotopic location and size of the SF-filtered stimuli.

In some (but not all) maps (e.g., Figures 3, 6, and 9), there was a partial overlap between place selectivity and high SF selectivity in PPA; the high SF effect was stronger in the posterior-lateral part of PPA. It is possible that PPA contains two functionally distinct sub-regions: (1) a “posterior-lateral” sub-region more influenced by low-level features and (2) an “anterior-medial” sub-region more influenced by cognitive factors. A similar subdivision of parahippocampal cortex has been reported previously [40],[41].

To resolve whether PPA responds to places versus lower-level spatial parameters such as SF, it would be ideal to quantitatively measure the relative strength of these two parameters in PPA. We did this test in monkeys (Figure 9) and in humans (Figure S7), and we obtained the qualitatively expected result. However, ultimately, it is not clear how to resolve this question quantitatively. How does one quantify “placeness”? What common metric can be used for both dimensions (i.e., what SF distribution equals how much placeness)? How does one deal with the known variation in acuity (and SF sensitivity) with retinal eccentricity? Further research is required to resolve these issues. Irrespective of the outcome, it should be simpler to interpret the functional processing in this intriguing higher-tier visual area, now that a robust and quantifiable lower-level stimulus selectivity has been identified.

Following the demonstration of face-selective regions in human visual cortex (e.g., [1]), it was shown that homologous face-selective regions also exist in visual cortex of macaque monkeys [2],[32]. This demonstration of homologous cortical regions across ∼50 million years of divergent primate evolution suggested that such neural mechanisms are fundamental to face processing, rather than some peculiarity of the human brain. More practically, the demonstration of face patches in monkeys made it possible to test the neural connections [42] and single-neuron response properties [3],[43] in the face patches, in ways that are impossible in human subjects. Here the demonstration of homologous “place areas” enables similar experimental opportunities for understanding the neural mechanisms underlying this intriguing system.

## Materials and Methods

### Subjects

Seven human subjects (with normal or corrected-to-normal vision) and three juvenile (5–7 kg) male rhesus monkeys (*Macaca mulatta*) were tested, in several experimental sessions each. Written informed consent was obtained from all human subjects prior to the scanning session. Surgical details and the training procedure for monkeys have been described elsewhere [33],[44]. All experimental procedures conformed to US National Institutes of Health guidelines and were approved by Massachusetts General Hospital protocols (#2000P-001155 for the human subjects, and #2005N-000201 for the monkeys). This research was conducted in accordance with the Weatherall report [45].

### Imaging Procedures and Data Analysis

All subjects were scanned in a horizontal 3T scanner (Siemens Tim Trio). Gradient echo EPI sequences were used for functional imaging (human: TR = 2,000 ms, TE  = 30 ms, flip angle = 90°, 3.0-mm isotropic voxels, and 33 axial slices; monkey: TR  = 2,000 ms, TE  = 19 ms, flip angle  = 90°, 1.0-mm isotropic voxels, and 50 axial slices). To increase functional magnetic resonance sensitivity in the monkey scans (to compensate for smaller voxels), we used a gradient insert coil (Siemens AC88), parallel imaging with a four-channel phased array coil, and an exogenous contrast agent (MION; 8–10 mg/kg IV). Throughout the functional scans, all subjects continuously fixated a small fixation spot at the center of the visual display, and eye position was monitored in monkeys using an infrared pupil tracking system (Iscan). A 3-D MP-RAGE sequence (1.0-mm isotropic voxels in humans; 0.35-mm isotropic voxels in anesthetized monkeys) was also used for high-resolution anatomical imaging from the same subjects. Functional and anatomical data were preprocessed and analyzed using FreeSurfer and FS-FAST (http://surfer.nmr.mgh.harvard.edu/).

For each subject, the inflated cortex and flattened cortical patches were reconstructed from MRI-based anatomical images. All functional images were motion corrected, spatially smoothed (unless otherwise noted) using a 3-D Gaussian kernel (2.5-mm half width at half maximum in humans and 1-mm half width at half maximum in monkeys), and normalized across scans. For intensity normalization, the BOLD image was first skull-stripped using the FSL (http://www.fmrib.ox.ac.uk/fsl/) Brain Extraction Tool to create a mask of brain-only voxels. Within this mask, the mean intensity of all voxels across all time points was computed. The value at each voxel at each time point was then scaled by 1,000 divided by the mean intensity, effectively forcing the mean in the mask to be 1,000.

The estimated hemodynamic response was defined by a γ function, and then the average signal intensity maps were calculated for each condition. Voxel-wise statistical tests were conducted by computing contrasts, based on a univariate general linear model. To avoid sampling gaps in the (high-resolution) monkey fMRI, we sampled and averaged the functional activity from all voxels located within the gray matter, along the surface normal. Finally, the significance levels were projected onto the inflated/flattened cortex after a rigid co-registration of functional and anatomical volumes. Functional maps were spatially normalized across sessions (in monkeys) and across subjects (in humans) using a spherical transformation, then averaged using a fixed-effects model.

### Visual Stimuli

Stimuli were generated on a PC (Windows XP) and presented via LCD projector (Sharp XG-P25, 1,024×768 pixels resolution, 60-Hz refresh rate) onto a rear-projection screen. The projector luminance was gamma-corrected using a hardware procedure. The gamma setting of the graphics card was set to the point closest to linearity. Additionally, the projector settings were adjusted to fine-tune the linear luminance transfer. The background luminance was set at 268 cd/m^2^. Matlab 7.0 and Psychophysics Toolbox (http://psychtoolbox.org/) were used to program the experiments.

The cube/sphere stimuli (∼12° in size), including “smooth,” “textured,” and “bumpy” versions, were all equated for surface reflectance, illumination, 3-D volume, and center location, and were presented on a random dot background.

The face/place stimuli (20° in size) were based on 20 grayscale face mosaic (“group photograph”) and place (indoor scene) images, which were qualitatively matched in visual complexity and natural image statistics (e.g., quantity of objects and degree of clutter) [46]. The initial face/place images (and also the black-white checkerboard images, which were 25° in size) were then spatially filtered (using a Hamming-windowed, causal linear-phase FIR filter with symmetric impulse response), and presented on a uniform gray background. The SF-filtered stimulus set included the following: low-pass (<1 c/deg for face; <1 c/deg or <0.41 c/deg for place; <0.5 c/deg for checkerboard), band-pass (1–5 c/deg for face/place; 0.5–5 c/deg for checkerboard), high-pass (>5 c/deg for face; >5 c/deg or >3.45 c/deg for place; >5 c/deg for checkerboard), and normal (unfiltered) images. Table 1 shows the root mean square contrast of these stimuli.

**Table 1:** The root mean square contrast of SF-filtered stimuli.

Spatial Frequency	Checkerboards	Faces	Places
Normal	0.50	0.21	0.18
High SF	0.08	0.05	0.04
Middle SF	0.29	0.11	0.09
Low SF	0.40	0.14	0.15

In each image class, the root mean square contrast values were averaged across all images in that class. In each image, the pixel intensities were normalized in the range [0,1].

The scene stimuli (20° in size, grayscale) were selected from a large scene image database (see Figure S10 for details).

All stimuli were presented in a blocked design. Within a functional run, the first and last blocks were null (fixation only) epochs, and the remaining stimulus blocks were ordered pseudo-randomly. In the cube/sphere experiments, the stimulus (e.g., a smooth cube) was presented unchanged throughout a given block (“single stimulus imaging”), except for the location of a virtual light source, which was systematically varied every second to refresh the fMRI activity (four source locations, ranging within ±32° in azimuth and ±25° in elevation). In the checkerboard experiments, the stimulus (e.g., high SF checks) was presented in different phases, within a fixed spatial envelope (the phase of the stimulus was systematically varied every 500 ms). In the face/place and scene experiments, multiple examples/images of a particular stimulus condition (e.g., high SF faces) were randomly presented within a given block, with each image presented for 1 s (in the face/place experiment) or 2 s (in the scene experiment).

To control for variations in attention, we had the human subjects detect/report a briefly presented red dot (0.2°×0.2°), which could appear anywhere on the cube/sphere shapes (Figures 1 and 2) and H/L scenes (Figure 7) during scanning at unpredictable times. Thus, to achieve a stable performance, subjects had to covertly attend to the full visuotopic extent of each stimulus. The detectability of the dot converged to 75% correct, based on a staircase modulation of dot luminance relative to the local background luminance (roughly, dot saturation). The subjects' performance was thus equated across different stimulus conditions. In the monkey experiments, attention was controlled by the fixation task, using juice reward.

Each human scan session consisted of 12 functional runs, with each run containing 18 blocks (block duration  = 16 s). Each monkey scan session consisted of 20 functional runs, with each run containing 10 blocks (block duration  = 24 s). In total, we collected 66,223 functional volumes (43 sessions) in humans, and 35,840 functional volumes (14 sessions) in monkeys.

## Supporting Information

Figure S1 **High-pass-filtered checkerboard images selectively activate PPA.** (A and B) An example of middle SF (A) and high SF (B) checkerboards. (C and D) The FFT of middle SF (C) and high SF (D) checkerboards. (E and F) The activity maps for faces versus places (E) and high SF (yellow/red) versus middle SF (cyan/blue) checkerboards (F), in the averaged human map. PPA responded selectively to the high SF checks. Other details are similar to those described in Figure 3.
(0.40 MB PDF)

Figure S2 **The PPA response to normal checkerboards.** (A and B) An example of the normal (unfiltered) checkerboard (A) and its FFT (B). The gray box around the checkerboard is for illustration purposes only. (C) The activity map for high SF versus normal checkerboards; the high SF checkerboard and its FFT are shown in Figure 3B and 3D. High-pass-filtered checks activated PPA significantly (*p* < 10^−2^) more than normal checks, even though normal checks contained a full range of SFs including high SF components. This suggests that the PPA activity is reduced in the presence of lower SFs, perhaps due to nonlinear suppressive interactions among the frequency channels (see Discussion). A similar effect can be seen in Figure S5 (the PPA response to SF-filtered faces).
(0.04 MB PDF)

Figure S3 **Region-of-interest analysis of checkerboard data in humans.** The bar plot shows the fMRI response to normal, high SF, middle SF, and low SF checkerboard stimuli in FFA and PPA. High SF checkerboards produced the highest fMRI response in PPA (*F*  = 4.15, *p* < 0.05; ANOVA, Sidak post-hoc test). Error bars indicate one standard error of the mean, based on a within-subjects ANOVA design.
(0.01 MB PDF)

Figure S4
**New SF-filtered place images, generated based on the power spectra of faces and places.** (A) First, the power spectra of ten group photo face images and ten place images were obtained, using a 2-D FFT. Then, the power spectra were averaged in each category. The averaged power spectra of faces and places were then converted to a 1-D plot, using rotational averaging. The plot is in “log-log” format. For each power spectrum (faces or places), one can define percent power in a given frequency range [f1, f2]: 
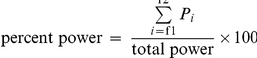
. The cut-off frequencies for faces were 1 c/deg for low-pass filtering (yellow dotted line) and 5 c/deg for high-pass filtering (yellow dashed line). The optimal cut-off frequencies for filtering places were defined in a way that low SF (and high SF) places had the same percent power as low SF (and high SF) faces. The calculated values were 0.41 c/deg for low-pass filtering (cyan dotted line) and 3.45 c/deg for high-pass filtering (cyan dashed line). (B) An example of high SF and low SF place stimuli, generated using these new cut-off frequencies.(0.05 MB PDF)

Figure S5 **fMRI response to SF-filtered faces.** The bar plot shows the percent signal change to normal, high SF, middle SF, and low SF face images in FFA and PPA. High SF faces produced a significantly higher activation in PPA compared to normal, middle SF, and low SF faces (*F* =  7.65, *p* < 0.05; ANOVA, Sidak post-hoc test). Error bars indicate one standard error of the mean, based on a within-subjects ANOVA design.
(0.01 MB PDF)

Figure S6 **fMRI response to SF-filtered places.** The bar plot shows the percent signal change to high SF and low SF place images in FFA and PPA (see Figure S4B for examples of stimuli). The asterisk denotes a statistically significant difference (*t* =  3.66, *p* < 0.01; paired *t*-test). Error bars indicate one standard error of the mean. PPA showed a higher fMRI response to high SF places (compared to low SF places).
(0.01 MB PDF)

Figure S7 **Relative strength of SF selectivity and place selectivity in human PPA.** In the left panel, the comparison between normal faces versus normal places revealed the location of FFA and PPA in the averaged map of seven human subjects. This group-averaged activity map is displayed on a ventral view of the averaged inflated cortical surface in the right hemisphere. In the comparison between high SF faces versus low SF places (middle panel), the high SF bias in PPA was strong enough that it essentially canceled the activity produced by places. As a control, the activity map for low SF faces versus high SF places (right panel) was virtually identical to the map of classical face/place localizer shown in the left panel. However, ultimately, the relative strength of these two variables cannot be quantified along a single common dimension, partly because “places” are ill-defined.
(0.03 MB PDF)

Figure S8 **Topographic representation of preferred SF in early (lower-tier) visual cortex.** Each panel shows an activity map on a flattened view of the occipital cortex, oriented in the right-hemisphere format for ease of comparison (left hemisphere from one representative human subject in the top row, and right hemisphere from three other human subjects in the bottom row). (A) The comparison between high SF (yellow/red) and middle SF (cyan/blue) conditions, collapsed across all face and place stimuli. (B and D–F) The comparison between high SF (yellow/red) and low SF (cyan/blue) conditions, collapsed across all face and place stimuli. (C) A conventional phase-encoded map of retinotopic eccentricity (e.g., [47]). It was produced by presenting black/white checkerboard rings at systematically varied visual field eccentricities. The eccentricity and preferred SF maps were qualitatively similar, but inversely related (see below) in early visual cortex (e.g., in V1, V2, and V3; their retinotopic/meridian borders are indicated with dotted and solid lines in [C]): a higher SF preference occurred at the representation of decreased retinotopic eccentricities (i.e., closer to the fovea, indicated with an asterisk).
(0.11 MB PDF)

Figure S9 **The location of high SF activity in PPA, relative to area V8/VO in human maps.** The comparison between responses to upper (cyan) and lower (yellow) visual field stimuli revealed area V8/VO in the ventral occipital cortex. This area contained both upper and lower visual field representations. The maps are displayed on a flattened view of human visual cortex (right hemisphere) in two representative subjects. The map threshold is *p* < 10^−3^. The black boundary indicates the location of high SF activity in the same subjects, based on the comparison between high SF and middle SF faces (see Figure 5). This activity was located anterior and ventral to V8/VO.
(0.16 MB PDF)

Figure S10 **Classifying natural scenes based on their power spectra.** For this classification, we used a large database of indoor and outdoor scenes (the TinyGraz03 dataset: http://www.emt.tugraz.at/~pinz/data/tinygraz03/). This database was originally intended for categorization of scenes from tiny (32 × 32 pixels) images [48]. The authors of the database provided us with the original 1,148 full-resolution images (all 512 × 512 pixels) from 20 different scene categories, ten indoor (e.g., living room, office, library) and ten outdoor (e.g., mountain, city, forest) categories. For each image, the high SF (SF > 5 c/deg) power was calculated. In order to compare the power values across images, the high SF power in an image was normalized by the total power in that image. (A) shows the percentage of the normalized high SF power for all images. The H1 and L1 images were the images that had the highest and the lowest normalized high SF power, respectively (see Figure 7A and 7B). The images were sorted/ranked based on their normalized high SF power values. After sorting, the first 50 images (with the highest rank) and the last 50 images (with the lowest rank) were classified as “H scenes” and “L scenes,” respectively. (B and C) show examples of H scenes (B) and L scenes (C). Eight images were randomly selected from each scene class to be used in a blocked-design fMRI experiment.
(0.67 MB PDF)

Figure S11 **The location of mPPA on the inflated cortical surface.** The activity map in the left panel shows mPPA and mFFA, based on the comparison between “group photo” faces versus places (see Materials and Methods). The activity map in the right panel shows mPPA and mFFA, based on a blocked-design comparison between large “single” faces (∼15° × 20° in size) versus places. As expected from the flattened cortical maps, mPPA was located immediately ventral to mFFA, as shown on a magnified view of macaque posterior IT cortex in the right hemisphere. The boundary of the “place” patch in the left panel was used in Figure 9F. PMTS, posterior middle temporal sulcus; OTS, occipito-temporal sulcus; STS, superior temporal sulcus.
(0.03 MB PDF)

Figure S12 **Evidence for mPPA in the subject average, using additional control stimuli.** In a separate experiment, we did a blocked-design comparison between places and single faces, objects, and body parts. In each stimulus block, multiple examples of each category were presented. The fMRI activity was measured in three macaque monkeys, and data from all monkeys were averaged using a random-effects model. The anatomical curvature pattern (underlay in the maps) was also averaged across subjects. The right hemisphere is shown on the left. The red blob of activity is mPPA, which responded significantly (*p* < 10^−2^) more to the place category than to the other categories. In both hemispheres, mPPA was topographically located lateral to the occipito-temporal sulcus (OTS), in caudal TE (posterior IT cortex).
(0.02 MB PDF)

Figure S13 **Topographic maps of SF sensitivity in macaque visual cortex.** The maps show the pattern of activity produced by high SF versus low SF checkerboards. The activity maps are displayed on a flattened view of macaque visual cortex (left hemisphere [LH] and right hemisphere [RH]). The white arrows indicate the high SF activity in the IT cortex, overlapping the mPPA (see Figure 9E and 9F). The maps also reflect the large-scale central-versus-peripheral bias in SF sensitivity (also shown in Figure S8 for human data), with an additional high SF extension into presumptive monkey TOS (see Figure 8). OTS, occipito-temporal sulcus; STS, superior temporal sulcus.
(0.35 MB PDF)

Figure S14 **Comparison between the FFT power spectra of building and face images.** In this analysis, we first computed the power spectra of ten building images and ten single face images (images commonly used in a PPA/FFA localizer). The power spectrum of each image was normalized by the power of the DC component. Then the building-versus-face spectral map was generated by subtracting the averaged power spectrum of buildings from the averaged power spectrum of faces (both in a decibel format). The red/blue color map represents the power (energy) difference in Fourier space (red/yellow: buildings have more energy than faces, particularly along horizontal and vertical orientations; blue/cyan: faces have more energy than buildings). Points near the center of the Fourier image correspond to low SFs. The single face images were selected from the Max Planck Institute for Biological Cybernetics Face Database (http://faces.kyb.tuebingen.mpg.de/), and the building images were selected from the Microsoft Research Cambridge Object Recognition Image Database (http://research.microsoft.com/en-us/downloads/b94de342-60dc-45d0-830b-9f6eff91b301/default.aspx).
(0.14 MB PDF)

## Abbreviations

c/deg: cycles/degree
FFA: fusiform face area
FFT: fast Fourier transform
fMRI: functional magnetic resonance imaging
IT: inferior temporal
mFFA: monkey homolog of fusiform face area
mPPA: monkey homolog of parahippocampal place area
PPA: parahippocampal place area
SF: spatial frequency
