# Supplementary figures and images for: The “Parahippocampal Place Area” Responds Preferentially to High Spatial Frequencies in Humans and Monkeys
Source: PLoS Biol. 2011 Apr 5;9(4):e1000608. doi: 10.1371/journal.pbio.1000608 (PMC3071373; doi:10.1371/journal.pbio.1000608)

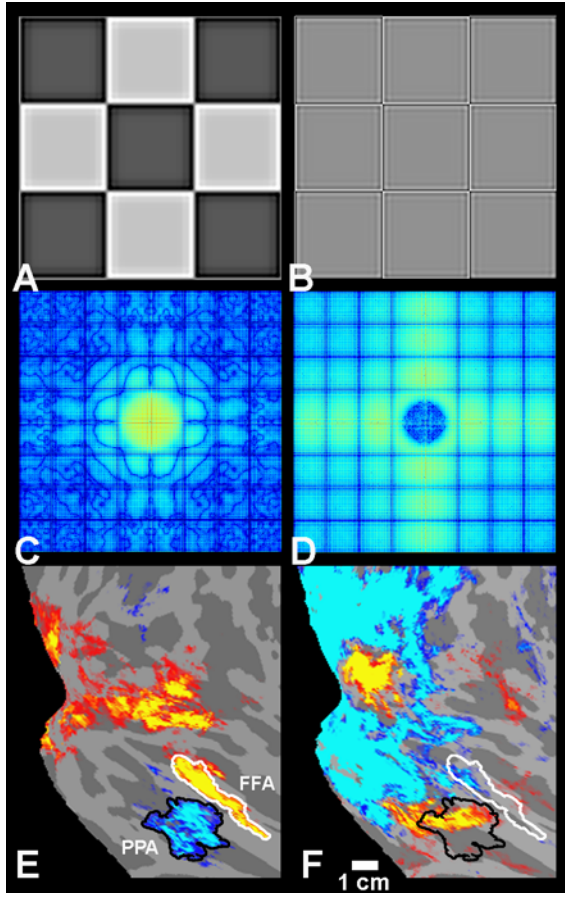

Supplement: Figure S1 — High-pass-filtered checkerboard images selectively activate PPA. (A and B) An example of middle SF (A) and high SF (B) checkerboards. (C and D) The FFT of middle SF (C) and high SF (D) checkerboards. (E and F) The activity maps for faces versus places (E) and high SF (yellow/red) versus middle SF (cyan/blue) checkerboards (F), in the averaged human map. PPA responded selectively to the high SF checks. Other details are similar to those described in Figure 3. (0.40 MB PDF) [file pbio.1000608.s001.pdf]

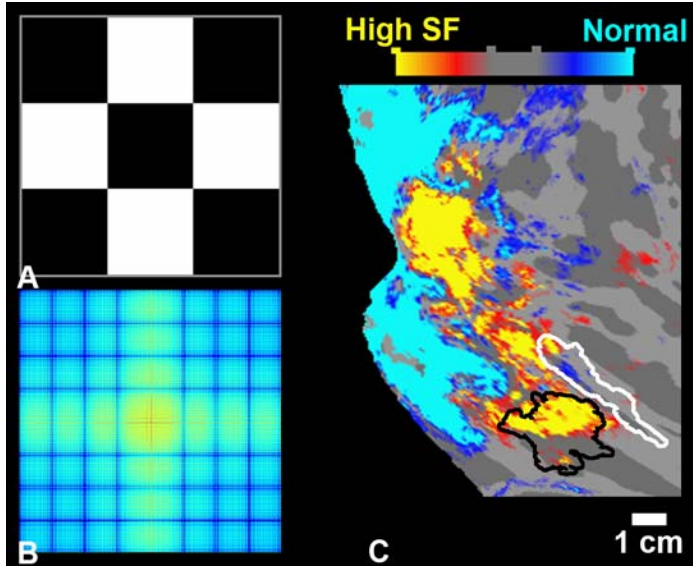

Supplement: Figure S2 — The PPA response to normal checkerboards. (A and B) An example of the normal (unfiltered) checkerboard (A) and its FFT (B). The gray box around the checkerboard is for illustration purposes only. (C) The activity map for high SF versus normal checkerboards; the high SF checkerboard and its FFT are shown in Figure 3B and 3D. High-pass-filtered checks activated PPA significantly (p < 10−2) more than normal checks, even though normal checks contained a full range of SFs including high SF components. This suggests that the PPA activity is reduced in the presence of lower SFs, perhaps due to nonlinear suppressive interactions among the frequency channels (see Discussion). A similar effect can be seen in Figure S5 (the PPA response to SF-filtered faces). (0.04 MB PDF) [file pbio.1000608.s002.pdf]

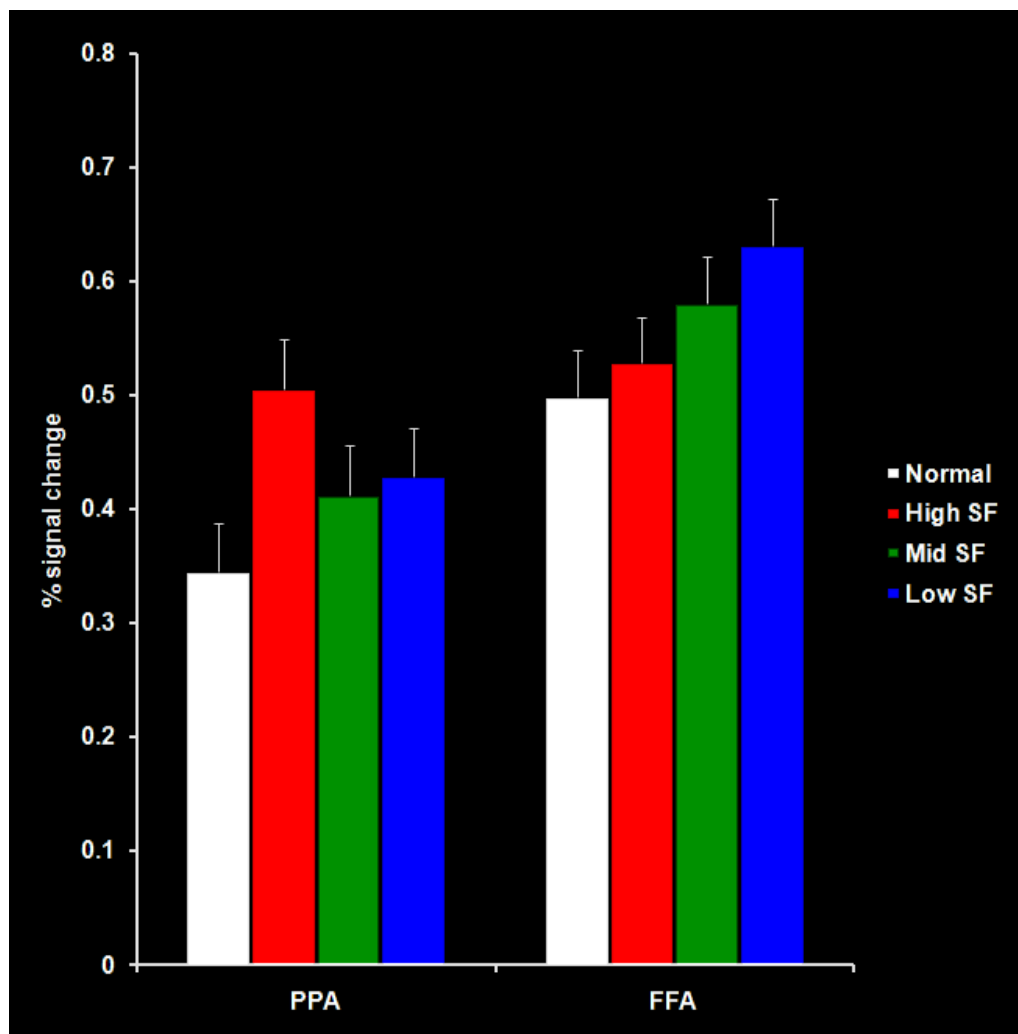

Supplement: Figure S3 — Region-of-interest analysis of checkerboard data in humans. The bar plot shows the fMRI response to normal, high SF, middle SF, and low SF checkerboard stimuli in FFA and PPA. High SF checkerboards produced the highest fMRI response in PPA (F = 4.15, p < 0.05; ANOVA, Sidak post-hoc test). Error bars indicate one standard error of the mean, based on a within-subjects ANOVA design. (0.01 MB PDF) [file pbio.1000608.s003.pdf]

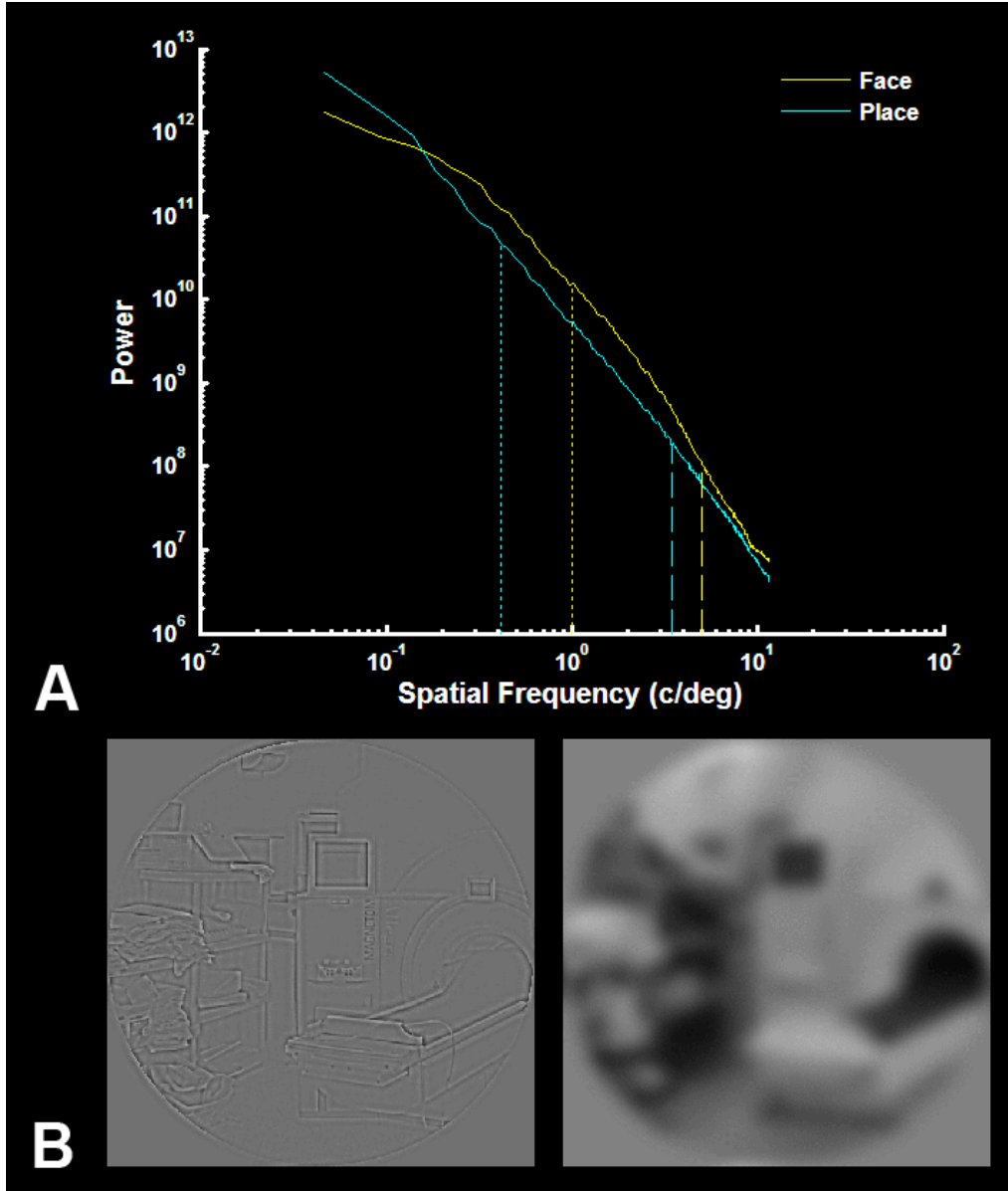

Supplement: Figure S4 — New SF-filtered place images, generated based on the power spectra of faces and places. (A) First, the power spectra of ten group photo face images and ten place images were obtained, using a 2-D FFT. Then, the power spectra were averaged in each category. The averaged power spectra of faces and places were then converted to a 1-D plot, using rotational averaging. The plot is in “log-log” format. For each power spectrum (faces or places), one can define percent power in a given frequency range [f1, f2]: . The cut-off frequencies for faces were 1 c/deg for low-pass filtering (yellow dotted line) and 5 c/deg for high-pass filtering (yellow dashed line). The optimal cut-off frequencies for filtering places were defined in a way that low SF (and high SF) places had the same percent power as low SF (and high SF) faces. The calculated values were 0.41 c/deg for low-pass filtering (cyan dotted line) and 3.45 c/deg for high-pass filtering (cyan dashed line). (B) An example of high SF and low SF place stimuli, generated using these new cut-off frequencies. (0.05 MB PDF) [file pbio.1000608.s004.pdf]

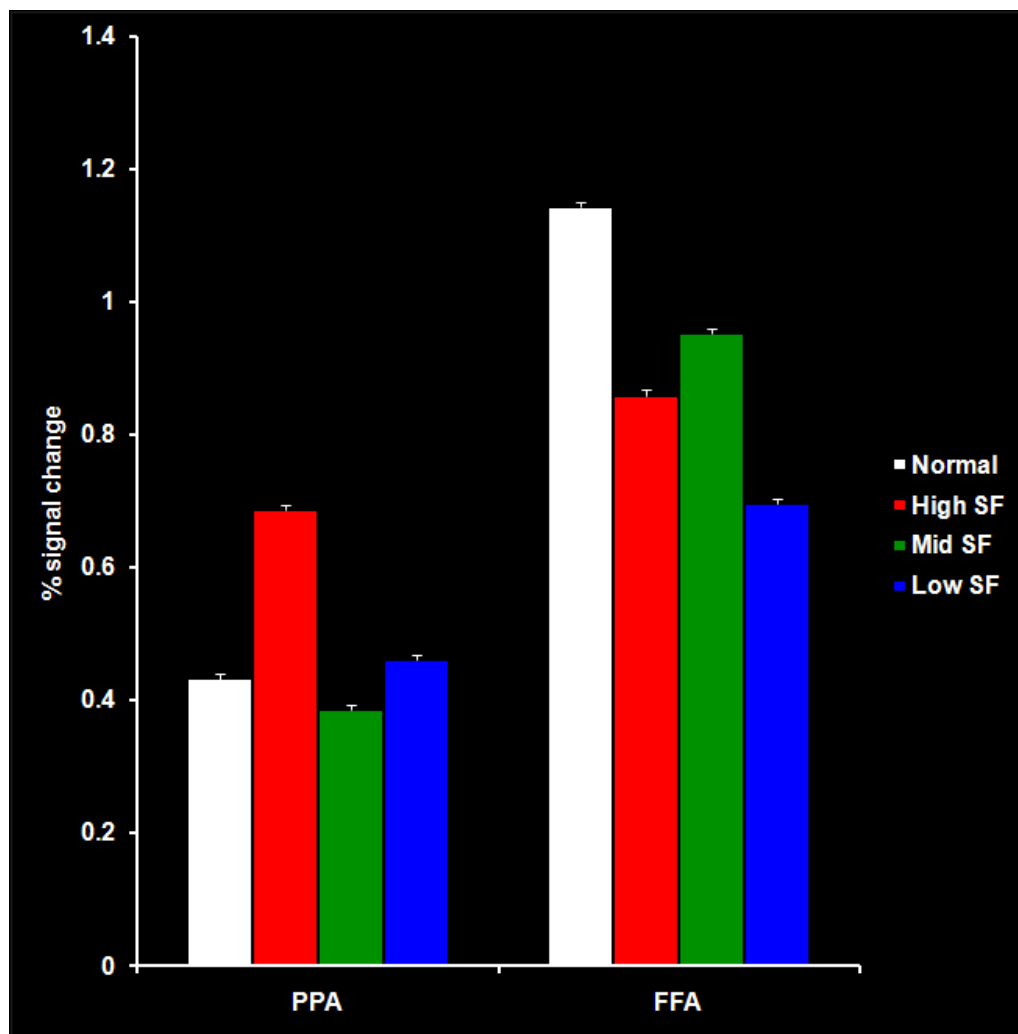

Supplement: Figure S5 — fMRI response to SF-filtered faces. The bar plot shows the percent signal change to normal, high SF, middle SF, and low SF face images in FFA and PPA. High SF faces produced a significantly higher activation in PPA compared to normal, middle SF, and low SF faces (F = 7.65, p < 0.05; ANOVA, Sidak post-hoc test). Error bars indicate one standard error of the mean, based on a within-subjects ANOVA design. (0.01 MB PDF) [file pbio.1000608.s005.pdf]

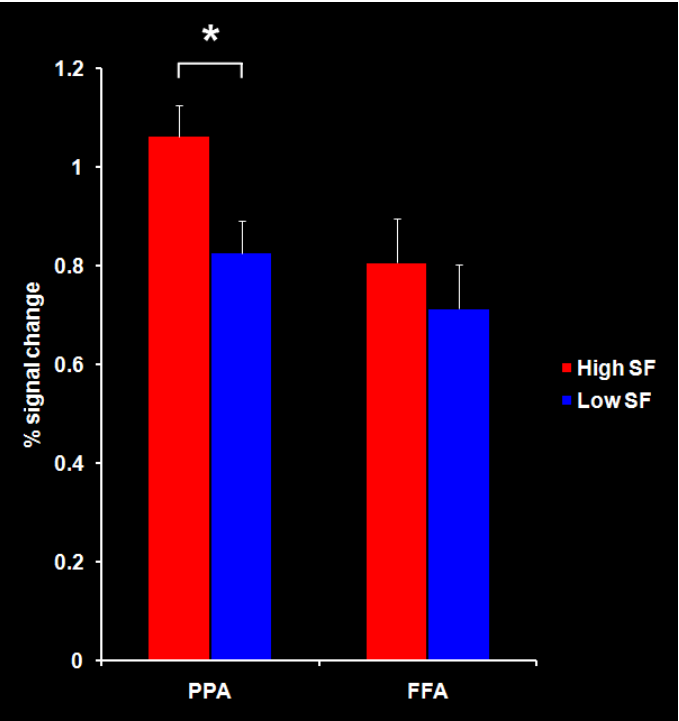

Supplement: Figure S6 — fMRI response to SF-filtered places. The bar plot shows the percent signal change to high SF and low SF place images in FFA and PPA (see Figure S4B for examples of stimuli). The asterisk denotes a statistically significant difference (t = 3.66, p < 0.01; paired t-test). Error bars indicate one standard error of the mean. PPA showed a higher fMRI response to high SF places (compared to low SF places). (0.01 MB PDF) [file pbio.1000608.s006.pdf]

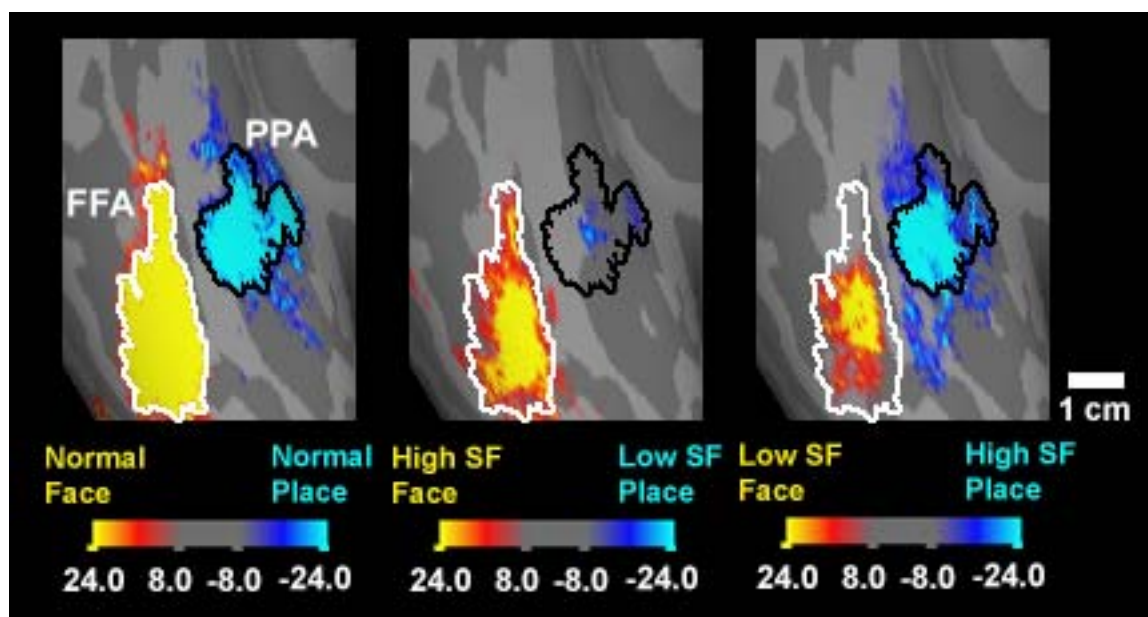

Supplement: Figure S7 — Relative strength of SF selectivity and place selectivity in human PPA. In the left panel, the comparison between normal faces versus normal places revealed the location of FFA and PPA in the averaged map of seven human subjects. This group-averaged activity map is displayed on a ventral view of the averaged inflated cortical surface in the right hemisphere. In the comparison between high SF faces versus low SF places (middle panel), the high SF bias in PPA was strong enough that it essentially canceled the activity produced by places. As a control, the activity map for low SF faces versus high SF places (right panel) was virtually identical to the map of classical face/place localizer shown in the left panel. However, ultimately, the relative strength of these two variables cannot be quantified along a single common dimension, partly because “places” are ill-defined. (0.03 MB PDF) [file pbio.1000608.s007.pdf]

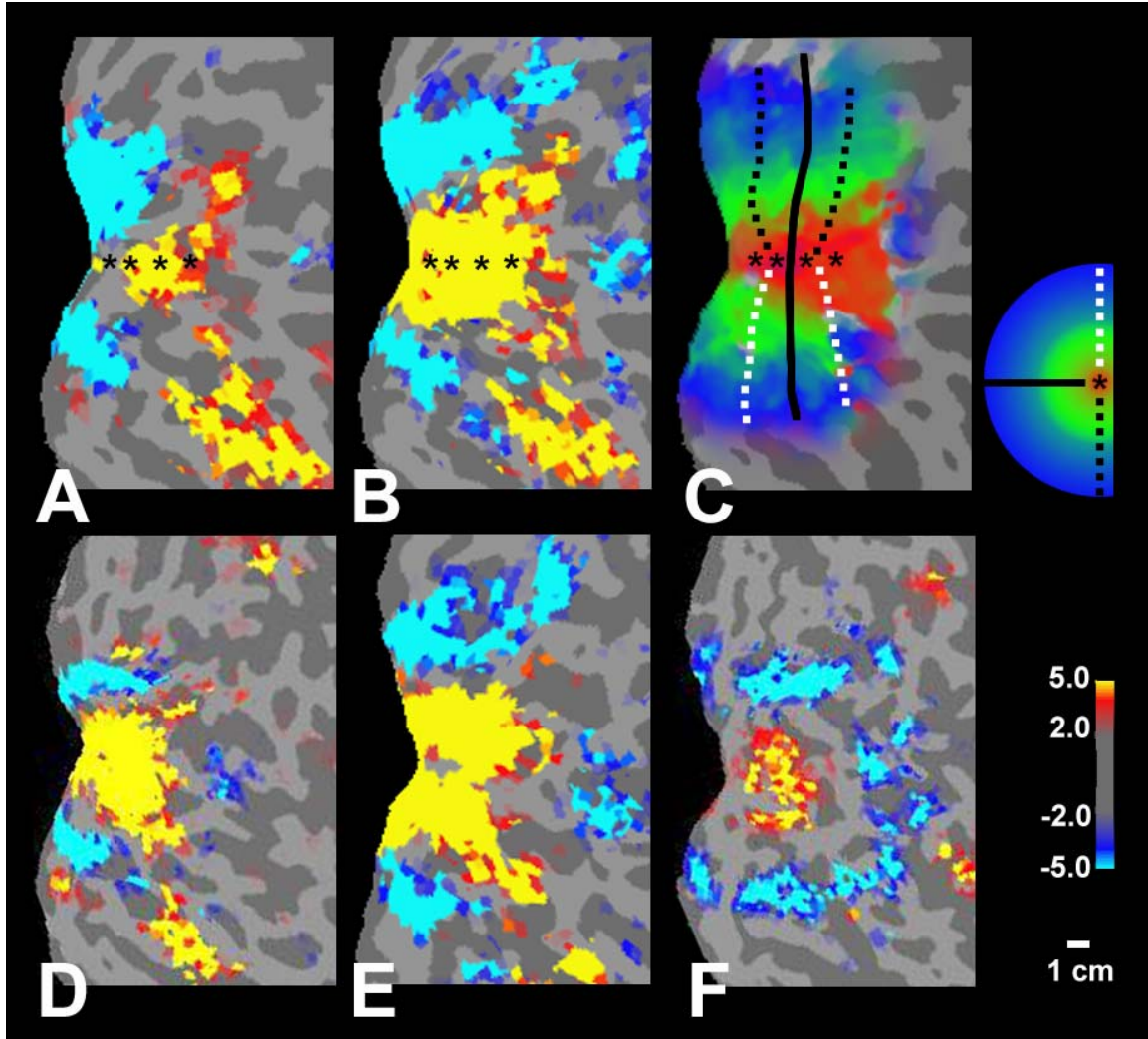

Supplement: Figure S8 — Topographic representation of preferred SF in early (lower-tier) visual cortex. Each panel shows an activity map on a flattened view of the occipital cortex, oriented in the right-hemisphere format for ease of comparison (left hemisphere from one representative human subject in the top row, and right hemisphere from three other human subjects in the bottom row). (A) The comparison between high SF (yellow/red) and middle SF (cyan/blue) conditions, collapsed across all face and place stimuli. (B and D–F) The comparison between high SF (yellow/red) and low SF (cyan/blue) conditions, collapsed across all face and place stimuli. (C) A conventional phase-encoded map of retinotopic eccentricity (e.g., [47]). It was produced by presenting black/white checkerboard rings at systematically varied visual field eccentricities. The eccentricity and preferred SF maps were qualitatively similar, but inversely related (see below) in early visual cortex (e.g., in V1, V2, and V3; their retinotopic/meridian borders are indicated with dotted and solid lines in [C]): a higher SF preference occurred at the representation of decreased retinotopic eccentricities (i.e., closer to the fovea, indicated with an asterisk). (0.11 MB PDF) [file pbio.1000608.s008.pdf]

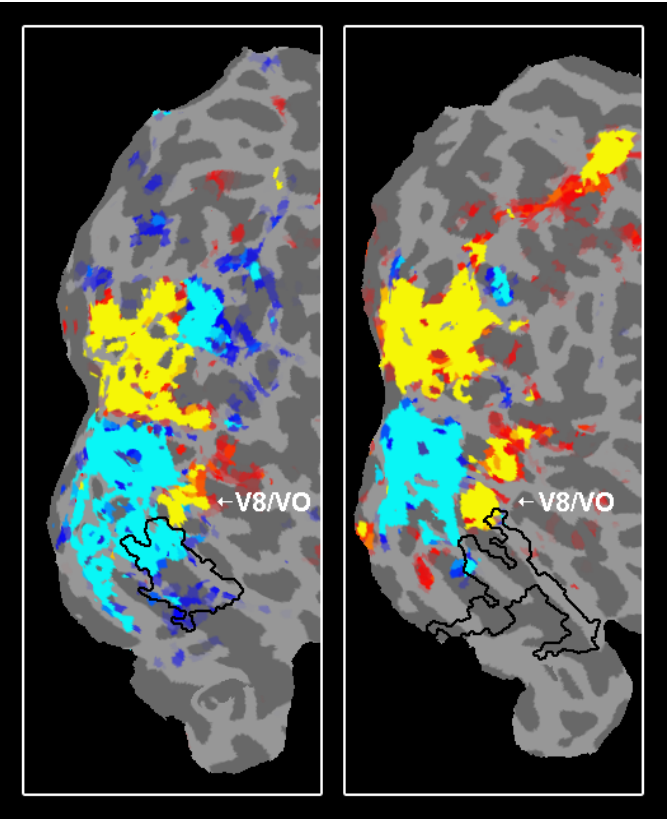

Supplement: Figure S9 — The location of high SF activity in PPA, relative to area V8/VO in human maps. The comparison between responses to upper (cyan) and lower (yellow) visual field stimuli revealed area V8/VO in the ventral occipital cortex. This area contained both upper and lower visual field representations. The maps are displayed on a flattened view of human visual cortex (right hemisphere) in two representative subjects. The map threshold is p < 10−3. The black boundary indicates the location of high SF activity in the same subjects, based on the comparison between high SF and middle SF faces (see Figure 5). This activity was located anterior and ventral to V8/VO. (0.16 MB PDF) [file pbio.1000608.s009.pdf]

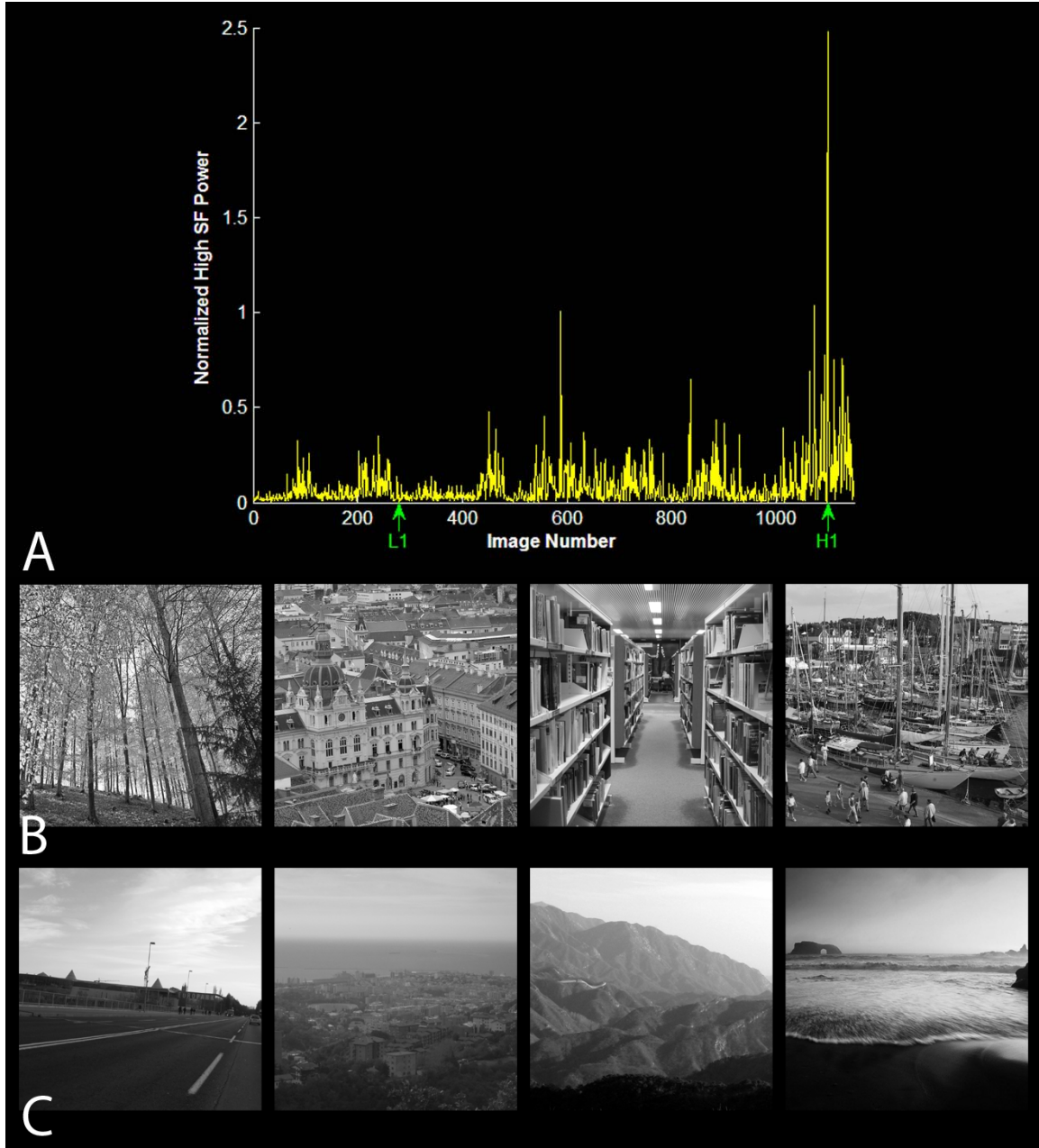

Supplement: Figure S10 — Classifying natural scenes based on their power spectra. For this classification, we used a large database of indoor and outdoor scenes (the TinyGraz03 dataset: http://www.emt.tugraz.at/~pinz/data/tinygraz03/). This database was originally intended for categorization of scenes from tiny (32 × 32 pixels) images [48]. The authors of the database provided us with the original 1,148 full-resolution images (all 512 × 512 pixels) from 20 different scene categories, ten indoor (e.g., living room, office, library) and ten outdoor (e.g., mountain, city, forest) categories. For each image, the high SF (SF > 5 c/deg) power was calculated. In order to compare the power values across images, the high SF power in an image was normalized by the total power in that image. (A) shows the percentage of the normalized high SF power for all images. The H1 and L1 images were the images that had the highest and the lowest normalized high SF power, respectively (see Figure 7A and 7B). The images were sorted/ranked based on their normalized high SF power values. After sorting, the first 50 images (with the highest rank) and the last 50 images (with the lowest rank) were classified as “H scenes” and “L scenes,” respectively. (B and C) show examples of H scenes (B) and L scenes (C). Eight images were randomly selected from each scene class to be used in a blocked-design fMRI experiment. (0.67 MB PDF) [file pbio.1000608.s010.pdf]

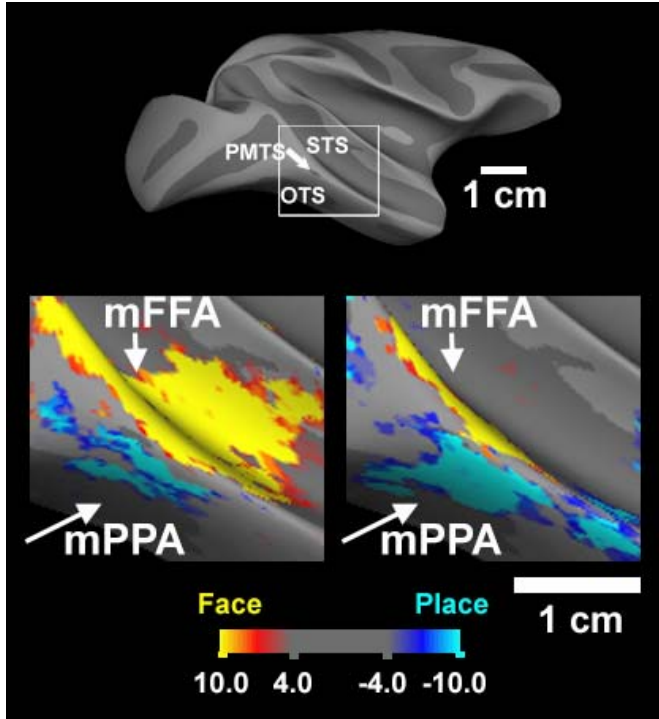

Supplement: Figure S11 — The location of mPPA on the inflated cortical surface. The activity map in the left panel shows mPPA and mFFA, based on the comparison between “group photo” faces versus places (see Materials and Methods). The activity map in the right panel shows mPPA and mFFA, based on a blocked-design comparison between large “single” faces (∼15° × 20° in size) versus places. As expected from the flattened cortical maps, mPPA was located immediately ventral to mFFA, as shown on a magnified view of macaque posterior IT cortex in the right hemisphere. The boundary of the “place” patch in the left panel was used in Figure 9F. PMTS, posterior middle temporal sulcus; OTS, occipito-temporal sulcus; STS, superior temporal sulcus. (0.03 MB PDF) [file pbio.1000608.s011.pdf]

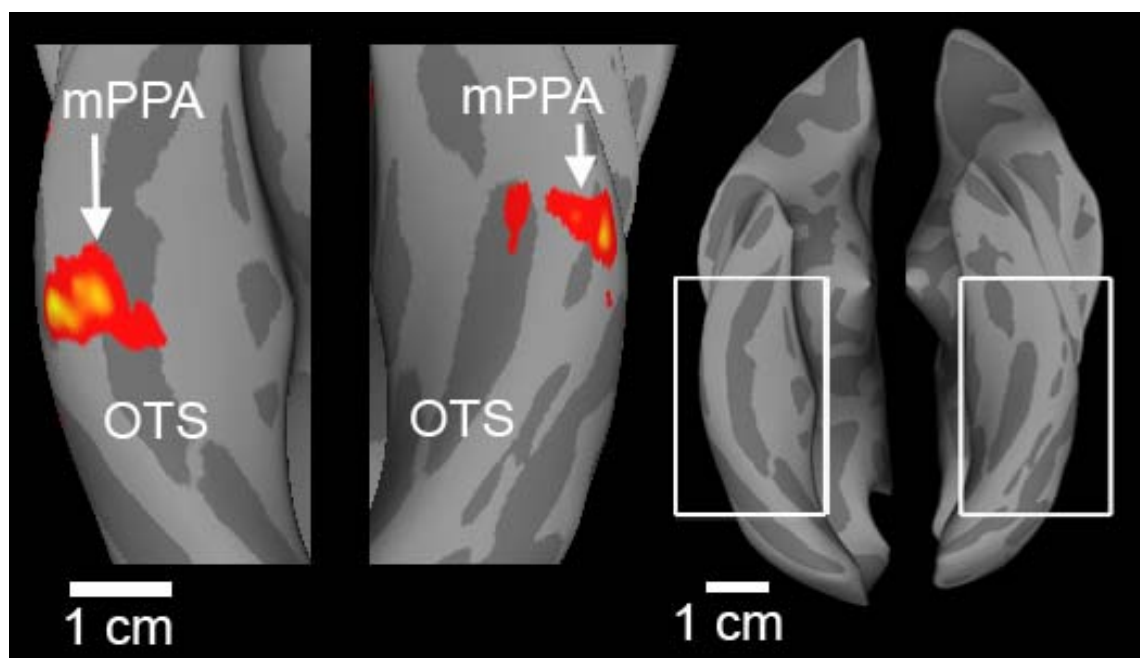

Supplement: Figure S12 — Evidence for mPPA in the subject average, using additional control stimuli. In a separate experiment, we did a blocked-design comparison between places and single faces, objects, and body parts. In each stimulus block, multiple examples of each category were presented. The fMRI activity was measured in three macaque monkeys, and data from all monkeys were averaged using a random-effects model. The anatomical curvature pattern (underlay in the maps) was also averaged across subjects. The right hemisphere is shown on the left. The red blob of activity is mPPA, which responded significantly (p < 10−2) more to the place category than to the other categories. In both hemispheres, mPPA was topographically located lateral to the occipito-temporal sulcus (OTS), in caudal TE (posterior IT cortex). (0.02 MB PDF) [file pbio.1000608.s012.pdf]

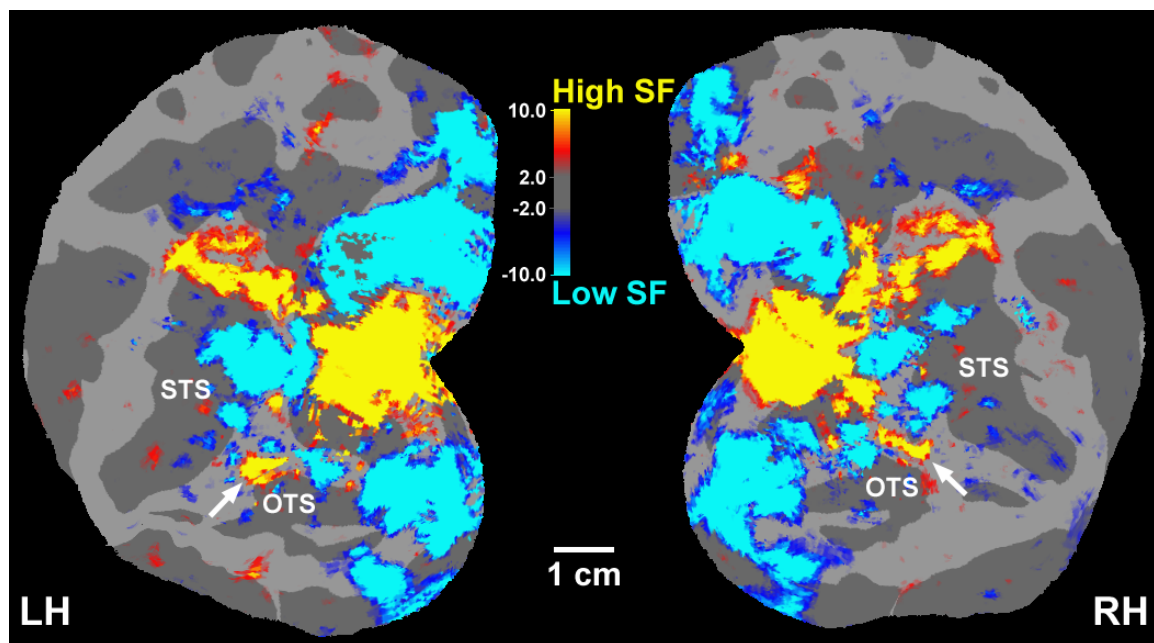

Supplement: Figure S13 — Topographic maps of SF sensitivity in macaque visual cortex. The maps show the pattern of activity produced by high SF versus low SF checkerboards. The activity maps are displayed on a flattened view of macaque visual cortex (left hemisphere [LH] and right hemisphere [RH]). The white arrows indicate the high SF activity in the IT cortex, overlapping the mPPA (see Figure 9E and 9F). The maps also reflect the large-scale central-versus-peripheral bias in SF sensitivity (also shown in Figure S8 for human data), with an additional high SF extension into presumptive monkey TOS (see Figure 8). OTS, occipito-temporal sulcus; STS, superior temporal sulcus. (0.35 MB PDF) [file pbio.1000608.s013.pdf]

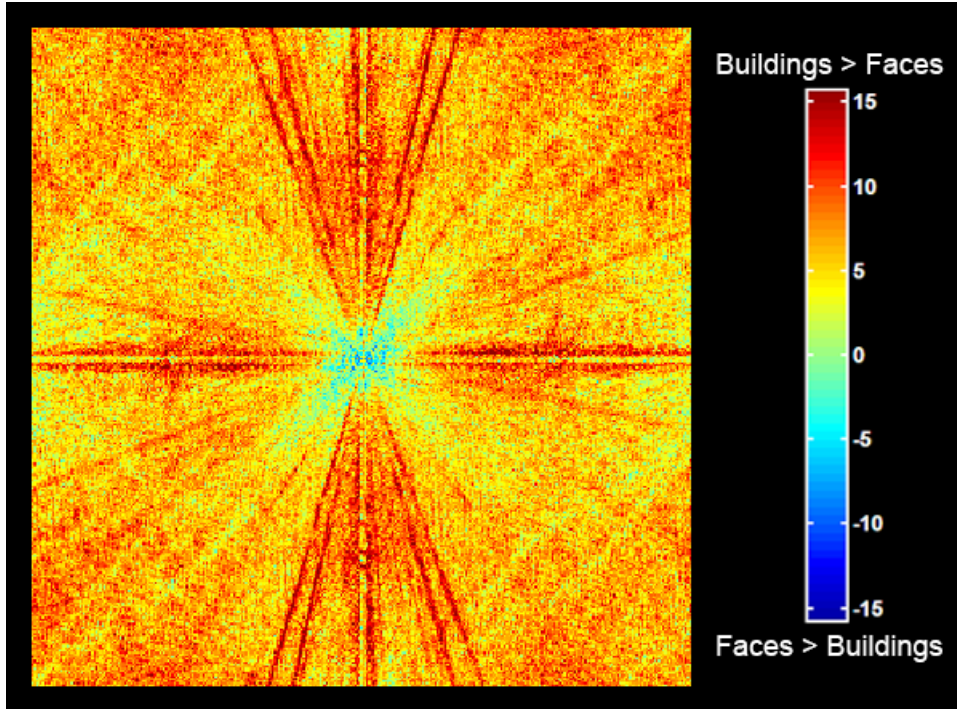

Supplement: Figure S14 — Comparison between the FFT power spectra of building and face images. In this analysis, we first computed the power spectra of ten building images and ten single face images (images commonly used in a PPA/FFA localizer). The power spectrum of each image was normalized by the power of the DC component. Then the building-versus-face spectral map was generated by subtracting the averaged power spectrum of buildings from the averaged power spectrum of faces (both in a decibel format). The red/blue color map represents the power (energy) difference in Fourier space (red/yellow: buildings have more energy than faces, particularly along horizontal and vertical orientations; blue/cyan: faces have more energy than buildings). Points near the center of the Fourier image correspond to low SFs. The single face images were selected from the Max Planck Institute for Biological Cybernetics Face Database (http://faces.kyb.tuebingen.mpg.de/), and the building images were selected from the Microsoft Research Cambridge Object Recognition Image Database (http://research.microsoft.com/en-us/downloads/b94de342-60dc-45d0-830b-9f6eff91b301/default.aspx). (0.14 MB PDF) [file pbio.1000608.s014.pdf]
